# Unbiased proteomic and forward genetic screens reveal that mechanosensitive ion channel MSL10 functions at ER–plasma membrane contact sites in *Arabidopsis thaliana*

**DOI:** 10.7554/eLife.80501

**Published:** 2022-10-07

**Authors:** Jennette M Codjoe, Ryan A Richardson, Fionn McLoughlin, Richard David Vierstra, Elizabeth S Haswell

**Affiliations:** 1 Department of Biology and the Center for Engineering Mechanobiology at Washington University in St. Louis St. Louis United States; University of Leeds United Kingdom; University of Freiburg Germany

**Keywords:** membrane contact sites, mechanotransduction, mechanosensitive ion channel, synaptotagmins, VAMP-associated proteins, *A. thaliana*

## Abstract

Mechanosensitive (MS) ion channels are an evolutionarily conserved way for cells to sense mechanical forces and transduce them into ionic signals. The channel properties of *Arabidopsis thaliana* MscS-Like (MSL)10 have been well studied, but how MSL10 signals remains largely unknown. To uncover signaling partners of MSL10, we employed a proteomic screen and a forward genetic screen; both unexpectedly implicated endoplasmic reticulum–plasma membrane contact sites (EPCSs) in MSL10 function. The proteomic screen revealed that MSL10 associates with multiple proteins associated with EPCSs. Of these, only VAMP-associated proteins (VAP)27-1 and VAP27-3 interacted directly with MSL10. The forward genetic screen, for suppressors of a gain-of-function *MSL10* allele (*msl10-3G, MSL10^S640L^*), identified mutations in the *synaptotagmin (SYT)5* and *SYT7* genes. We also found that EPCSs were expanded in leaves of *msl10-3G* plants compared to the wild type. Taken together, these results indicate that MSL10 associates and functions with EPCS proteins, providing a new cell-level framework for understanding MSL10 signaling. In addition, placing a mechanosensory protein at EPCSs provides new insight into the function and regulation of this type of subcellular compartment.

## Introduction

Eukaryotic cells have evolved multiple mechanisms to coordinate responses between cellular compartments (Schrader et al., 2015; Mielecki et al., 2020; Sampaio et al., 2022). One such mechanism is the formation of membrane contact sites—subcellular locations where membranes of two organelles are held in close proximity by tethering proteins—which serve as sites of exchange, signaling, and organization in all eukaryotic cells (Scorrano et al., 2019; Prinz et al., 2020). One type of membrane contact site is the enfdoplasmic reticulum (ER)–plasma membrane (PM) contact site (EPCS). Mammalian EPCSs are important sites for the metabolism and transport of phospholipids and allow for the coordination of ion fluxes (Zaman et al., 2020; Li et al., 2021). In plants, EPCSs help maintain phospholipid homeostasis and cell integrity (Schapire et al., 2008; Ruiz-Lopez et al., 2021), are hubs of endocytosis (Stefano et al., 2018) and autophagy (Wang et al., 2019), and regulate cell–cell transport at plasmodesmata (Levy et al., 2015; Ishikawa et al., 2020).

Several components of plant EPCSs are conserved across eukaryotes. The integral ER proteins synaptotagmins (SYTs) and vesicle-associated membrane protein (VAMP)-associated protein (VAP)27s are homologous to tricalbins and Scs2/Scs22, respectively, in yeast, and to extended-synaptotagmins and VAPs, respectively, in mammals. In yeast, tricalbins and Scs2 and Scs22 additively contribute to tethering the ER and PM to each other (Manford et al., 2012), and it is likely that plant SYTs and VAP27s also have a cooperative tethering function. Plant VAP27s may serve as a scaffold as they are known to interact with a variety of proteins and link EPCSs to endocytic (Stefano et al., 2018) and autophagic (Wang et al., 2019) machinery as well as to the actin and microtubule cytoskeletons (Wang et al., 2014; Zang et al., 2021). Plant SYTs are required to maintain plasma membrane integrity in the face of stressors (Schapire et al., 2008; Yamazaki et al., 2008; Pérez-Sancho et al., 2015; Ruiz-Lopez et al., 2021), probably by transporting lipids between the ER and PM (Qian et al., 2022) like their yeast and mammalian counterparts (Saheki et al., 2016; Qian et al., 2021). Furthermore, *Arabidopsis thaliana* SYT1 changes localization and is required for cell integrity in response to mechanical pressure (Pérez-Sancho et al., 2015), implicating EPCSs in the perception of mechanical stimuli. However, how mechanical information might be transmitted to or from EPCSs is completely unknown.

Organisms have evolved a variety of strategies to sense and respond to mechanical stimuli. One kind of mechanosensory protein—the mechanosensitive (MS) ion channel—represents a particularly ancient strategy that most cells still use (Arnadóttir and Chalfie, 2010; Booth et al., 2015). Most MS ion channels open and conduct ions in response to lateral membrane tension, transducing mechanical stimuli like touch, vibration, swelling, or shearing into an electrochemical signal (Kefauver et al., 2020). There is some understanding of the stimuli that activate particular plant MS channels (cell swelling, cell shrinking, encountering a barrier) as well as the adaptive processes in which they participate (relieving cell swelling, enhancing salinity tolerance, root penetration, regulating organellar morphology) (Codjoe et al., 2022). What is less understood is how signals from MS channels are coordinated across cell compartments and transduced to trigger longer-term, plant-level adaptations.

*Arabidopsis* MscS-Like (MSL)10 is a member of a conserved family of MS channels found in plants, bacteria, archaea, and some fungi (Hamilton et al., 2015). MSL10 is a bona fide MS ion channel and its tension-sensitive channel properties are relatively well-characterized (Haswell et al., 2008; Maksaev and Haswell, 2012; Maksaev et al., 2018). MSL10 is plasma membrane-localized (Haswell et al., 2008; Veley et al., 2014), and genetic studies have implicated it in a range of physiological roles. In response to hypo-osmotic cell swelling, MSL10 promotes a cytosolic Ca^2+^ transient, the accumulation of reactive oxygen species, the induction of *TOUCH* gene expression, and programmed cell death (Basu and Haswell, 2020a). MSL10 also contributes to systemic electrical and Ca^2+^ signaling in response to wounding (Moe-Lange et al., 2021). *MSL10* gain-of-function lines—including *MSL10-GFP* overexpressors (Veley et al., 2014) and the EMS-induced point mutant *msl10-3G* (Zou et al., 2016)—lead to constitutive growth inhibition and ectopic cell death (Basu et al., 2020b) through a pathway that requires the immune co-chaperone SGT1b/RAR1/HSP90 complex, although this is likely far downstream of MSL10 activation (Basu et al., 2022). Earlier events in signal transduction by MSL10 remain largely unknown.

MSL10 has primarily been studied at the protein level or at the whole plant level, but its function at the subcellular level has not been addressed. To understand how MSL10 transduces mechanical information into whole-plant phenotypes, we searched for potential signaling partners through proteomic and forward genetic screens. Here, we describe how both approaches, in combination with live-imaging assays, reveal that MSL10 functions at EPCSs.

## Results

### Immunoprecipitation–mass spectrometry to identify the MSL10 interactome

**Figure 1.**
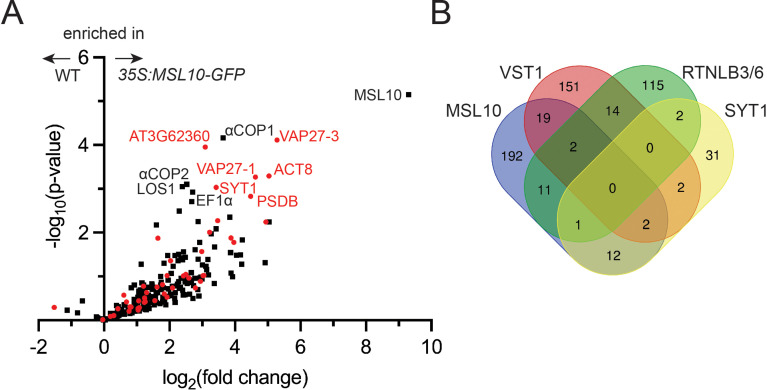
Co-immunoprecipitation–liquid chromatography-tandem mass spectrometry (LC-MS/MS) identifies the MSL10-GFP interactome, which shares similarities to previous endoplasmic reticulum–plasma membrane contact site (EPCS) interactomes. (**A**) Volcano plot showing the abundance of proteins detected in immunoprecipitations of MSL10-GFP in *35S:MSL10-GFP* seedlings (right) compared to those identified in mock immunoprecipitations using WT Col-0 seedlings (left). Proteins were identified by LC-MS/MS, and the average abundance of each was quantified from the MS1 precursor ion intensities. Only those proteins with at least eight peptide spectral matches are shown. Each protein is plotted based on its -log_10_(p-value) of significance based on four biological replicates relative to its log_2_(fold change) of abundance (*35S:MSL10-GFP/* WT). Proteins also detected in immunoprecipitations with the EPCS proteins SYT1 (Ishikawa et al., 2020), VST1 (dataset filtered for proteins with >8 peptide-spectral matches [PSMs]; Ho et al., 2016), and VAP27-1/3 (Stefano et al., 2018) or plasmodesmata-associated RTNLB3/6 (Kriechbaumer et al., 2015) are represented as red circles; proteins unique to the MSL10 interactome are represented as black squares. The 11 most significantly enriched proteins are labeled (p-value<0.002). (**B**) The overlap of the indicated interactomes with that of MSL10. The VAP27-1/3 interactome (Stefano et al., 2018) was not included here because only eight selected interactors were reported. Figure 1—source data 1.Peptide abundances from LC-MS/MS from mock, MSL10-GFP, and MSL10 7D-GFP immunoprecipitations.

We first searched for signaling partners that physically interact with MSL10 using an unbiased proteomic approach. Here, GFP-tagged MSL10, which has the same electrophysiological and cell death signaling properties as untagged MSL10 (Maksaev and Haswell, 2012; Basu et al., 2020b), was used as bait for immunoprecipitation–mass spectrometry. Microsomes were isolated from seedlings expressing *35S:MSL10-GFP* (Veley et al., 2014) and MSL10-GFP was immunoprecipitated from solubilized microsome extracts using GFP-Trap beads. Liquid chromatography-tandem mass spectrometry (LC-MS/MS) was performed on four replicate immunoprecipitations from *35S:MSL10-GFP* seedlings as well as four mock immunoprecipitations from WT (Col-0) microsomes. In total, we identified 1904 peptides that mapped to 606 protein groups in the MSL10-GFP-enriched samples, 239 proteins of which had at least 8 peptide spectral matches (Figure 1—source data 1). As shown in the volcano plot reporting enrichment and significance (Figure 1A), a number of proteins were identified as significantly enriched in MSL10-GFP pull-downs. Most of the proteins identified were also pulled down with MSL10^7D^-GFP, an inactive version of MSL10 wherein seven serines presumed to be phosphorylation sites were mutated to aspartate or glutamate (Veley et al., 2014; Basu et al., 2020b; Figure 1—figure supplement 1A), suggesting that the interactions were not dependent on MSL10 cell death-inducing activity. In fact, no detected proteins had significantly altered abundance (fold change > 4 and p-value<0.05) in the MSL10 compared to MSL10^7D^ proteomes (Figure 1—figure supplement 1B).

**Figure 1—figure supplement 1.**
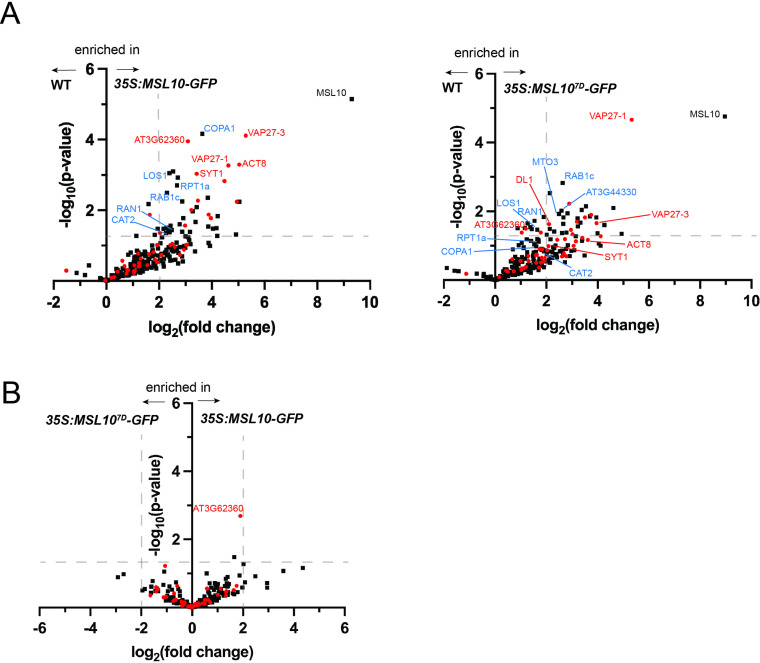
Similar proteins were identified in MSL10-GFP and MSL10^7D^-GFP immunoprecipitations. (**A**) Volcano plots showing the preferential abundance of proteins detected in immunoprecipitations of *35S:MSL10-GFP* (left, reproduced from Figure 1A) or *35S:MSL10^7D^-GFP* (right) compared to those of mock immunoprecipitations of WT Col-0 seedlings. (**B**) displays the relative abundance of proteins in *35S:MSL10-GFP* (right) vs. *35S:MSL10^7D^-GFP* (left) immunoprecipitations. Proteins were identified by liquid chromatography-tandem mass spectrometry (LC-MS/MS), and the average abundance of each was quantified from the MS1 precursor ion intensities, and only those proteins with at least eight peptide spectral matches are shown. Each protein is plotted based on its -log_10_(p-value) of significance based on four biological replicates relative to its log_2_(fold change) of abundance. Data points indicated as red circles have previously detected in interactomes of SYT1 (Ishikawa et al., 2020), VAP27-1/3 (Stefano et al., 2018), RTNLB3/6 (Kriechbaumer et al., 2015), and VST1 (Ho et al., 2016). Labeled are proteins that were selected for further testing in Figure 2A, selected because in either the MSL10-GFP and/or MSL10^7D^-GFP co-immunoprecipitations they were above the cutoffs indicated as dashed gray lines: fold change > 4 and p-values<0.05. Those with red labels have been found previously in endoplasmic reticulum–plasma membrane contact site (EPCS) or plasmodesmatal interactomes, and those in blue have not.

Among the most enriched proteins in the MSL10-GFP pulldowns were VAP27-1, VAP27-3/PVA12, and SYT1/SYTA, each of which is a known component of plant EPCSs (Levy et al., 2015; Wang et al., 2014; Stefano et al., 2018). The peptides detected covered over 30% of the full-length protein sequence for MSL10, VAP27-1, and VAP27-3; and over 11% of the protein sequence for SYT1 (Figure 1—source data 1). The interactome list led us to perform a meta-analysis comparing the proteins that co-immunoprecipitated with MSL10 or MSL10^7D^ with three previously published interactomes generated with established EPCS components: SYT1 (Ishikawa et al., 2020), VAP-RELATED SUPPRESSOR OF TMM 1 (VST1) (Ho et al., 2016), and VAP27-1 and VAP27-3 (Stefano et al., 2018), as well as an interactome of reticulon-like proteins RTNLB3 and RTNLB6, ER-shaping proteins found at plasmodesmata that interact with SYT1 and VAP27s (Kriechbaumer et al., 2015). Twenty percent of the proteins that co-immunoprecipitated with MSL10-GFP were detected in at least one of these EPCS interactomes, strongly suggesting that MSL10 interacts with EPCSs (Figure 1A, shown in red). For example, of the 10 proteins most enriched in the MSL10-GFP pulldowns (other than MSL10, the bait), five were previously known to be associated with plant EPCSs: SYT1, VAP27-1, VAP27-3, actin 8 (ACT8), and AT3G62360 (a predicted protein with a carbohydrate binding-like fold). Although no single protein was detected in all interactomes compared, MSL10 shared 23 interacting proteins with VST1, 15 with SYT1, and 14 with RTNLB3/6 (Figure 1B). These interactomes may only partially overlap because they are incomplete, because protein complexes at EPCSs are large and difficult to fully survey, and/or because there are different EPCS complexes in different cell types or in different conditions. Nevertheless, these results indicat that MSL10 physically associates with protein complexes located at EPCSs.

### MSL10 directly interacts with VAP27-1 and VAP27-3

**Figure 2.**
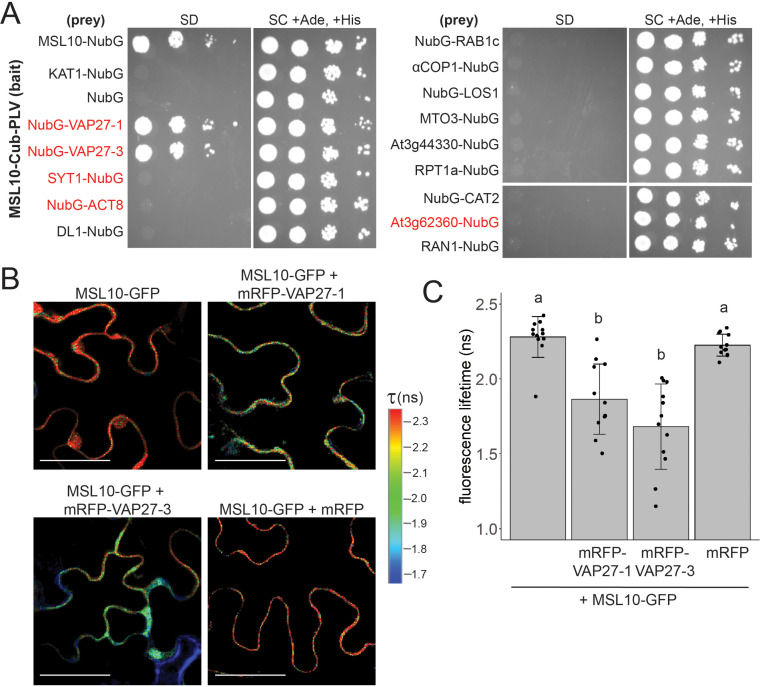
MSL10 interacts with VAP27-1 and VAP27-3. (**A**) Mating-based split-ubiquitin (mbSUS) assay. VAMP-associated protein 27-1 (VAP27-1), VAP27-3, synaptotagmin 1 (SYT1), actin 8 (ACT8), dynamin-like (DL1), RAB GTPase homolog 1c (RAB1c), coatomer α1 subunit (αCOP1), LOW EXPRESSION OF OSMOTICALLY RESPONSIVE GENES 1 (LOS1), METHIONINE OVERACCULATOR 3 (MTO3), AT3G44330, regulatory particle triple-A 1A (RPT1a), catalase 2 (CAT2), AT3G62360, and Ras-related nuclear protein 1 (RAN1) were fused to NubG and tested for interaction with Cub-tagged MSL10. Proteins labeled in red were previously detected at endoplasmic reticulum–plasma membrane contact sites (EPCSs). The results in (**A**) are consistent with a second independent mbSUS assay using independent transformants. (**B, C**) In vivo Förster resonance energy transfer–fluorescence lifetime imaging microscopy (FRET-FLIM) on *UBQ:MSL10-GFP* and *UBQ:mRFP-VAP27-1* or *UBQ:mRFP-VAP27-3* transiently expressed in tobacco. (**B**) Representative heat maps of the fluorescence lifetime (τ) of GFP measured in tobacco abaxial epidermal cells 5 days post-infiltration. Scale = 50 µm. (**C**) Average GFP fluorescence lifetime. Each data point represents the value from one field of view (three fields of view per plant from four infiltrated plants for a total of n = 12 for each combination). Error bars, SD. Groups indicated by the same letter are not statistically different according to ANOVA with Tukey’s post-hoc test.

We next asked whether MSL10 directly interacts with a subset of its proteome. We selected 14 of the 38 most highly enriched proteins from MSL10-GFP and/or MSL10^7D^-GFP pulldowns (fold change > 4 and p-value<0.05), including the five previously associated with EPCSs, for further testing. These five proteins included At3g62360, which was enriched in the MSL10-GFP pulldowns compared to MSL10^7D^-GFP, though at levels below the selected cutoff (Figure 1—figure supplement 1B). We first employed the yeast mating-based split-ubiquitin system (mbSUS) (Obrdlik et al., 2004; Figure 2A). MSL10 (the bait) and the candidate interactors (the prey) were tagged with the C- and N-terminal halves of ubiquitin, respectively, using orientations whereby each tag was predicted to face the cytosol. As previously reported, MSL10-Cub was able to interact with MSL10-NubG but did not interact with the potassium channel KAT1-NubG or untagged NubG (Basu et al., 2020b). Of the 14 tested yeast strains, only those expressing NubG-VAP27-1 and NubG-VAP27-3 survived on minimal media when mated to yeast expressing MSL10-Cub. Consistent with our proteomic results (Figure 1—figure supplement 1B), the interaction between MSL10 and VAP27s in the split-ubiquitin assay was not appreciably altered when the inactive MSL10^7D^ phosphovariant was used as bait (Figure 2—figure supplement 1A). The interaction was also maintained when using the overactive MSL10^7A^ (Veley et al., 2014; Basu et al., 2020b) or MSL10^S640L^ (*msl10-3G*; Zou et al., 2016) variants, suggesting that the activation of MSL10 signaling does not alter its ability to interact with VAP27-1 and VAP27-3. Furthermore, the conserved major sperm protein domains of VAP27s were not required for interaction with MSL10 (Figure 2—figure supplement 1B). Along with the absence of known VAP27-binding motifs (James and Kehlenbach, 2021) in MSL10, these results indicate that MSL10 interacts with VAP27-1 and VAP27-3 in a non-canonical way.

**Figure 2—figure supplement 1.**
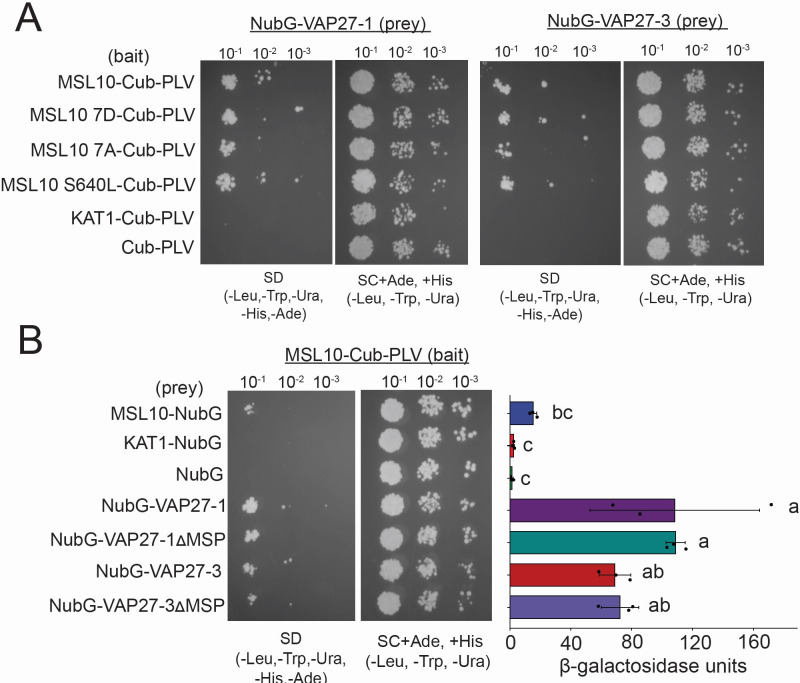
MSL10 signaling mutants interact with VAP27-1 and VAP27-3, and the VAP27 MSP domain is dispensable for interaction. Mating-based split-ubiquitin assays testing (**A**) the interaction of full-length VAP27-1 and VAP27-3 with mutant versions of full-length MSL10 and (**B**) the interaction of full-length MSL10 with variants of VAP27-1 and VAP27-3 that lacked their major sperm protein (MSP) domain (VAP27-1∆6-125 and VAP27-3∆23-142). MSL10^7A^ is a phosphodead variant in which the seven phosphoserines in the N-terminus of MSL10 are mutated to alanine (Veley et al., 2014; Basu et al., 2020b). The MSL10^S640L^ substitution (*msl10-3G* allele) occurs in the cytosolic C-terminal domain of MSL10 (Zou et al., 2016). Both variants trigger constitutive overactivation of MSL10 cell death signaling. At right in (**B**): measurements of β-galactosidase activity in a liquid-based assay testing the same combinations at left using CPRG as substrate.

We employed Förster resonance energy transfer–fluorescence lifetime imaging microscopy (FRET-FLIM) to provide additional evidence that MSL10 directly interacts with VAP27-1 and VAP27-3 in plant cells. In FRET-FLIM, when proteins are close enough for energy transfer (<10 nm), the fluorescence lifetime of the FRET donor decreases (Sun et al., 2012). MSL10-GFP transiently expressed in tobacco leaves had a fluorescence lifetime of 2.3 ± 0.1 ns (Figure 2B and C). When co-expressed with mRFP-VAP27-1 or mRFP-VAP27-3, MSL10-GFP lifetimes were 1.8 ± 0.2 ns (a 22% decrease) and 1.6 ± 0.3 ns (a 30% decrease), respectively. Co-expressing MSL10-GFP and free mRFP did not alter the fluorescence lifetime of GFP. These fluorescence lifetimes with and without acceptors are in the same range as those previously reported for interactions between proteins expressed in tobacco (Wang et al., 2014; Wang et al., 2019).

### A subpopulation of MSL10 co-localizes with a subpopulation of VAP27-1 and VAP27-3

**Figure 3.**
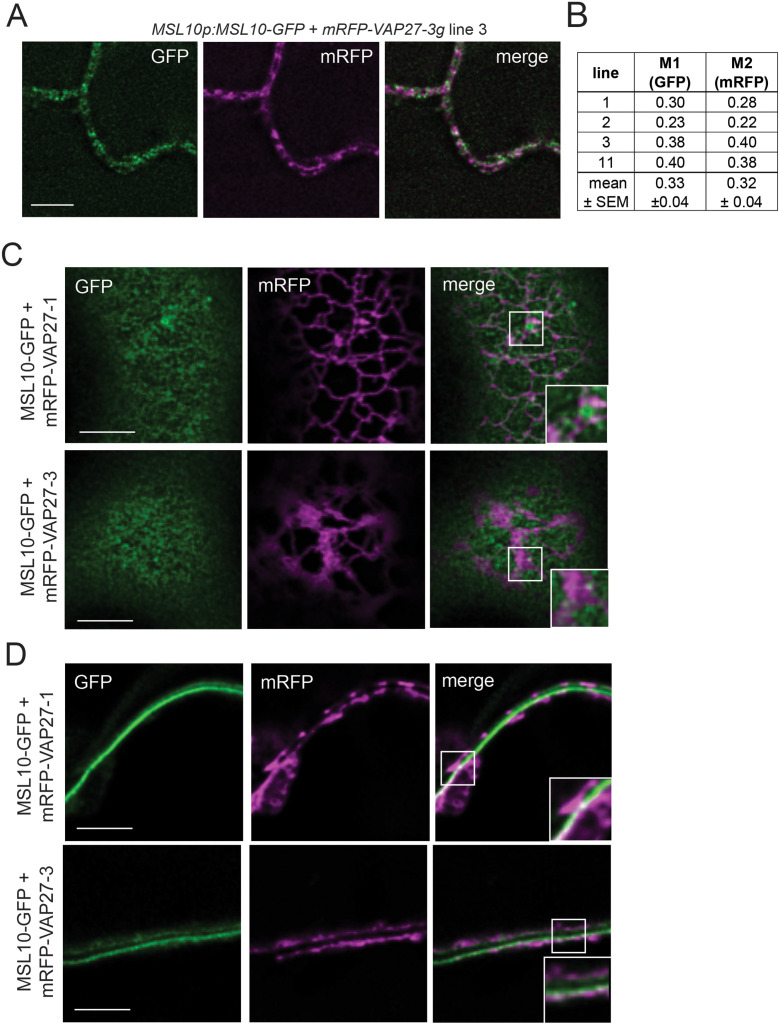
A subpopulation of MSL10 co-localizes with a subpopulation of VAP27-1 and VAP27-3. (**A**) Equatorial deconvolved confocal laser scanning micrographs of leaf abaxial epidermal cells from stable *Arabidopsis* T1 lines co-expressing MSL10-GFP and mRFP-VAP27-3 driven by their endogenous promoters. Scale = 5 µm. (**B**) Mander’s overlap coefficients M1 and M2 calculated from images taken from four independent T1 lines. (**C, D**) Deconvolved confocal micrographs showing a Z-slice at the top (cortical, **C**) and the middle (equatorial, **D**) of tobacco epidermal cells transiently expressing *UBQ:MSL10-GFP* and *UBQ:mRFP-VAP27-1* or *UBQ:mRFP-VAP27-3.* Images were taken 5 days after infiltration. Scale = 5 µm.

To support our observation that MSL10 and VAP27s interact, we sought evidence in stable transgenic *A. thaliana* lines expressing *MSL10-GFP* and *mRFP-VAP27-3* under the control of their respective promoters. We examined localization in leaf epidermal cells, where EPCSs are commonly studied and *MSL10* and *VAP27-3* are expressed (eFP Browser; Winter et al., 2007). As expected, MSL10-GFP displayed a punctate localization at the periphery of leaf epidermal cells (Figure 3A; Veley et al., 2014; Maksaev et al., 2018). In four independent *MSL10p:MSL10-GFP+mRFP-VAP27-3g* lines, mRFP signal was punctate at the cell periphery and only partially co-localized with GFP signal. On average, across the four lines, 33 ± 4% of MSL10-GFP signal co-localized with mRFP-VAP27-3 in equatorial images, while 32 ± 4% of mRFP-VAP27-3 co-localized with MSL10-GFP (Mander’s overlap coefficient M1 and M2, respectively, Figure 3B). Due to low endogenous expression of MSL10-GFP and cell wall autofluorescence, we could not obtain a cortical image of MSL10-GFP and mRFP-VAP27-3 co-localization in *Arabidopsis*. Instead, we examined co-localization in cortical and equatorial slices of tobacco leaf epidermal cells transiently overexpressing MSL10-GFP and mRFP-VAP27-3 or mRFP-VAP27-1 (Figure 3C and D). These images confirm what we observed in *Arabidopsis*—that only a subpopulation of MSL10 co-localized with VAP27s, and vice versa. This is similar to what has been observed with the PM-localized aquaporin *Zm*PIP2;5 and *Zm*VAP27-1 (Fox et al., 2020). Additionally, the majority of MSL10-GFP, even when overexpressed, trafficked to the plasma membrane, whereas mRFP-VAP27-1 and mRFP-VAP27-3 were found in the ER just below.

Taken together, the data shown in Figures 1—3 indicate that a subpopulation of MSL10 interacts directly with two VAP27s and indirectly with several other components of EPCSs. Because VAP27-1 and VAP27-3 are integral ER proteins (Saravanan et al., 2009; Wang et al., 2014) and MSL10 is found in the plasma membrane (Haswell et al., 2008; Veley et al., 2014), an interaction between the two would, by definition, create an EPCS.

### MSL10 alters EPCS morphology by expanding SYT1 puncta

**Figure 4.**
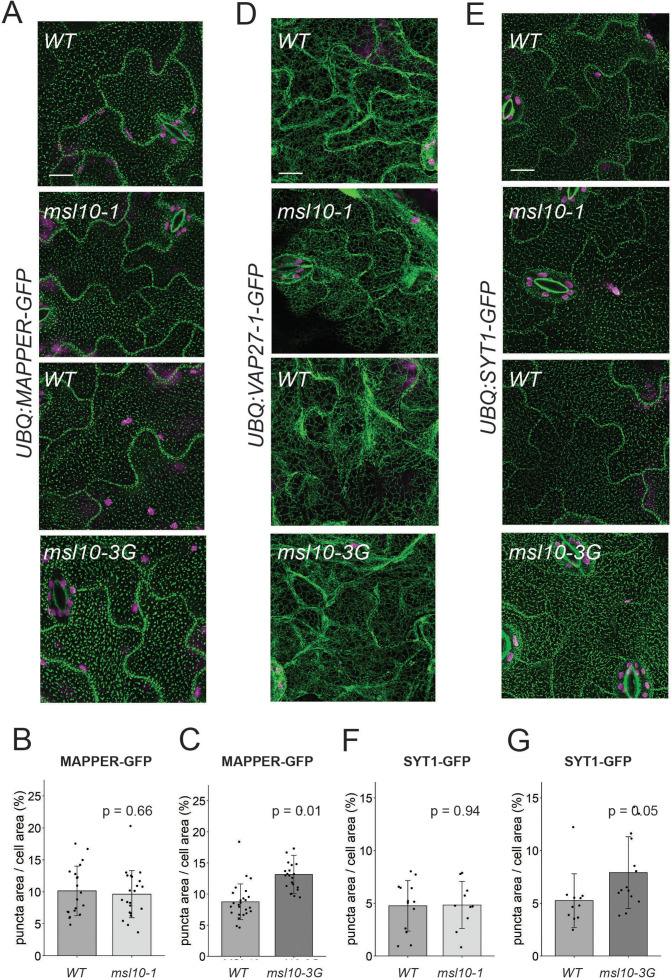
Some endoplasmic reticulum–plasma membrane contact sites (EPCSs) are expanded in *msl10-3G* plants. Confocal Z-projections (maximum intensity projection of Z-slices from the top to the middle of cells) of GFP-tagged proteins in the indicated *MSL10* backgrounds. MAPPER-GFP (**A**), VAP27-1-GFP (**D**), and SYT1-GFP (**E**) in 4-week-old abaxial leaf epidermal cells. Plants shown here are cousins (**A, E**) or siblings (**D**). Green, GFP; magenta, chlorophyll autofluorescence. Scale = 10 µm. Quantification of the percentage of the leaf epidermal cell volume taken up by MAPPER-GFP (**B, C**) or SYT1-GFP (**F, G**) puncta in plants in the *msl10-1* or *msl10-3G* background compared to WT cousins. Each data point represents a biological replicate: the mean value of 20–50 epidermal cells from one plant, n = 10–25 plants per genotype from two or three separately grown flats. Error bars, SD. Means were compared by Student’s *t*-tests when data was normally distributed (**B, F**) or Mann–Whitney *U*-tests when it was not (**C, G**).

Given that EPCS patterning is stress-responsive (Pérez-Sancho et al., 2015; Lee et al., 2019; Lee et al., 2020; Ruiz-Lopez et al., 2021), we hypothesized that MSL10 might serve a regulatory function at EPCSs. We began to test this hypothesis by investigating the effect of *MSL10* mutant alleles on the localization of a general EPCS marker, Membrane-Attached PeriPhERal (MAPPER)-GFP (Chang et al., 2013). We crossed a *UBQ:MAPPER-GFP* line (Lee et al., 2019) to loss-of-function (*msl10-1;* Haswell et al., 2008) and gain-of-function (*msl10-3G*; Zou et al., 2016; Basu et al., 2020b) mutant plant lines. In the F3 generation, we compared MAPPER-GFP localization in WT, *msl10-1,* or *msl10-3G* backgrounds. MAPPER-GFP puncta looked similar in segregated WT and *msl10-1* plants (Figure 4A and B). In contrast, MAPPER-GFP puncta were expanded in adult *msl10-3G* plants (Figure 4A and C), taking up a larger proportion (13.1 ± 3.1%) of the cellular area in adult *msl10-3G* leaf epidermal cells compared to those in plants with the WT *MSL10* allele (8.7 ± 2.9%).

**Figure 4—figure supplement 1.**
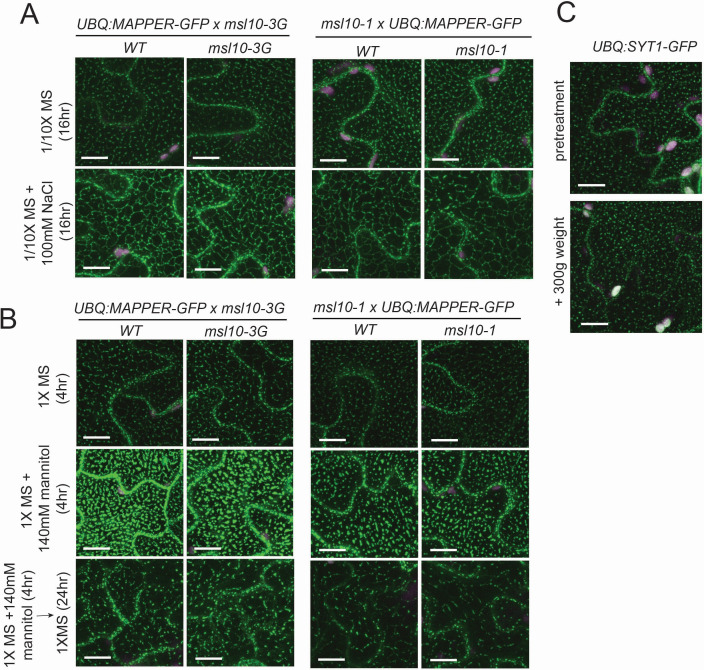
MSL10 does not influence rearrangements in endoplasmic reticulum–plasma membrane contact sites (EPCS) morphology in response to osmotic stress in seedlings. Confocal maximum intensity Z-projections of cotyledon epidermal cells. Green, GFP; magenta, chlorophyll autofluorescence. Scale = 10 µm. The seedlings being compared in (**A, B**) are F3 cousins. (**A**) Five-day-old seedlings were transferred from plates and incubated for 16 hr in liquid 1/10× MS or 1/10× MS + 100 mM NaCl. (**B**) Seedlings were grown on 1× MS or 1× MS +140 mM mannitol plates for 5 days. Seedlings were transferred to liquid media of the same concentration supplemented with 600 nM isoxaben and allowed to equilibrate for 4 hr before imaging (as described in Basu and Haswell, 2020a). MAPPER-GFP puncta size is larger in the presence of mannitol, in contrast to observations by Lee et al., 2019, and the difference might be attributable to the presence of mannitol in the plates for the entire life of the seedlings, used here. A subset of seedlings incubating in 1× MS + 140 mM mannitol + 600 nM isoxaben were transferred to liquid 1× MS + 600 nM isoxaben to trigger cell swelling, and these were imaged 24 hr later. (**C**) Five-day-old seedlings stably expressing *UBQ:SYT1-GFP* were mounted in water. Cotyledons were imaged before and after a 300 g weight was applied to a 22 × 22 mm coverslip for 20 s.

We next examined VAP27 and SYT1 localization. We generated lines stably expressing VAP27-1-GFP, VAP27-3-GFP, and SYT1-GFP under control of the *UBQ10* promoter and crossed them to *msl10-1* and *msl10-3G* plants. The genotypes of surviving F2 seedlings from some of these crosses indicated genetic interactions between *MSL10* and the overexpression transgenes. For example, we were unable to isolate plants carrying the *UBQ:VAP27-3-GFP* transgene in either the *msl10-1* or *msl10-3G* homozygous backgrounds when grown on soil, and fewer *msl10-1; UBQ:SYT1-GFP* plants were isolated than would be predicted by normal Mendelian segregation (Table 1).

**Table 1.** Segregation of MSL10 alleles in crosses to lines overexpressing GFP-labelled endoplasmic reticulum–plasma membrane contact sites (EPCS) proteins. msl10-1 and msl10-3G plants were crossed to lines expressing GFP-labelled VAP27-1, VAP27-3, SYT1, SYT5, and SYT7 under the control of the UBQ10 promoter. F2 plants (or F3 offspring of heterozygous F2 plants) were selected based on Basta resistance driven by the UBQ:GFP transgenes, and resistant plants were genotyped for the indicated MSL10 alleles. Chi-squared tests were calculated based on a predicted 1:2:1 segregation ratio. Crosses that had significant deviations (Pp<0.05) from expected ratios are in bold.

		# Basta resistant offspring with indicated genotypes				
Parental genotype		MSL10/MSL10	MSL10/msl10-3G	msl10-3G/msl10-3G	X^2^	P
UBQ:VAP27-1-GFP/-;	MSL10/msl10-3G	6/25 (24%)	16/25 (64%)	3/25 (12%)	2.68	0.26
**UBQ:VAP27-3-GFP/-;**	**MSL10/msl10-3G**	**12/33 (36%)**	**21/33 (64%)**	**0/33 (0%)**	**11.18**	**0.004**
UBQ:SYT1-GFP/-;	MSL10/msl10-3G	6/21 (29%)	12/21 (57%)	3/21 (14%)	1.29	0.53
UBQ:SYT5-GFP/-;	MSL10/msl10-3G	5/21 (24%)	7/21 (33%)	9/21 (43%)	3.86	0.15
UBQ:SYT7-GFP/-;	MSL10/msl10-3G	9/40 (23%)	23/40 (57%)	8/40 (20%)	0.95	0.62
		MSL10/MSL10	MSL10/msl10-1	msl10-1/msl10-1		
UBQ:VAP27-1-GFP/-;	MSL10/msl10-1	6/28 (21%)	17/28 (61%)	5/28 (18%)	1.36	0.51
**UBQ:VAP27-3-GFP/-;**	**MSL10/msl10-1**	**7/36 (19%)**	**29/36 (81%)**	**0/36 (0%)**	**16.17**	**0.0003**
**UBQ:SYT1-GFP/-;**	**MSL10/msl10-1**	**24/74 (33%)**	**46/74 (62%)**	**4/74 (5%)**	**15.19**	**0.0005**
UBQ:SYT5-GFP/-;	MSL10/msl10-1	7/23 (30%)	8/23 (35%)	8/23 (35%)	2.22	0.33
UBQ:SYT7-GFP/-;	MSL10/msl10-1	16/42 (38%)	17/42 (41%)	9/42 (21%)	3.86	0.15
**Expected ratios**		**25%**	**50%**	**25%**		

VAP27-1-GFP is localized to the ER in *Arabidopsis* leaf epidermal cells, forming some puncta (although fewer than reported for VAP27-1 when transiently overexpressed in tobacco; Wang et al., 2014; Wang et al., 2016). We found that the VAP27-1 localization pattern was similar in *msl10-1, msl10-3G,* and their segregated WT *MSL10* backgrounds (Figure 4D). As there were so few VAP27-1-GFP puncta, we did not quantify their area as for MAPPER-GFP. Due to the presumed synthetic lethality described above, we were unable to assess the effect of MSL10 on VAP27-3 EPCSs. SYT1-GFP displayed the expected punctate localization (Levy et al., 2015; Pérez-Sancho et al., 2015), and SYT1-GFP localization was unchanged in the *msl10-1* background (Figure 4E and F). However, in the *msl10-3G* background, SYT1-GFP puncta were expanded in leaf epidermal cells compared to the WT, leading to a modest, but significant increase in SYT1-GFP area relative to cellular area (Figure 4E and G). This SYT1-GFP pattern closely resembled that observed with the MAPPER-GFP marker (compare Figure 4A and D).

### MSL10 does not contribute to EPCS rearrangement in response to osmotic perturbations

SYT-EPCSs are sensitive to environmental conditions, quickly changing localization in response to mechanical pressure (Pérez-Sancho et al., 2015) and slowly remodeling in response to freezing and salinity stress and the presence of rare ions (Lee et al., 2019; Lee et al., 2020; Ruiz-Lopez et al., 2021). We tested whether MSL10 was required for some of these EPCS rearrangements. As previously reported (Lee et al., 2019), EPCSs marked by MAPPER-GFP in cotyledon epidermal cells expanded after a 16 hr exposure to 100 mM NaCl (Figure 4—figure supplement 1A). A similar MAPPER-GFP localization pattern was also observed in *msl10-1* and *msl10-3G* seedlings treated with NaCl, indicating that MSL10 does not influence the expansion of EPCSs during salinity stress. Salinity-induced EPCS expansion is reversible when seedlings are moved to media lacking NaCl, triggering a hypo-osmotic shock (Lee et al., 2019). As MSL10 plays a role in the cellular response to hypo-osmotic cell swelling (Basu and Haswell, 2020a), we asked whether MSL10 was also responsible for EPCS shrinking under these conditions. We found that MAPPER-GFP signal decreased in cotyledon epidermal cells 24 hr after hypo-osmotic shock (Figure 4—figure supplement 1B) but that this phenomenon was unaffected by the *msl10-1* or *msl10-3G* alleles. SYT1-GFP has been reported to move from a ‘beads on a string’ localization pattern to a punctate one when mechanical stress is applied (Pérez-Sancho et al., 2015). In our hands, SYT1-GFP localization always appeared punctate in cotyledon epidermal cells, and we did not see an appreciable change in this localization when pressure was added (Figure 4—figure supplement 1C).

### A forward genetic screen provides evidence for functional interactions between *MSL10* and *SYT5* and *SYT7*

**Figure 5.**
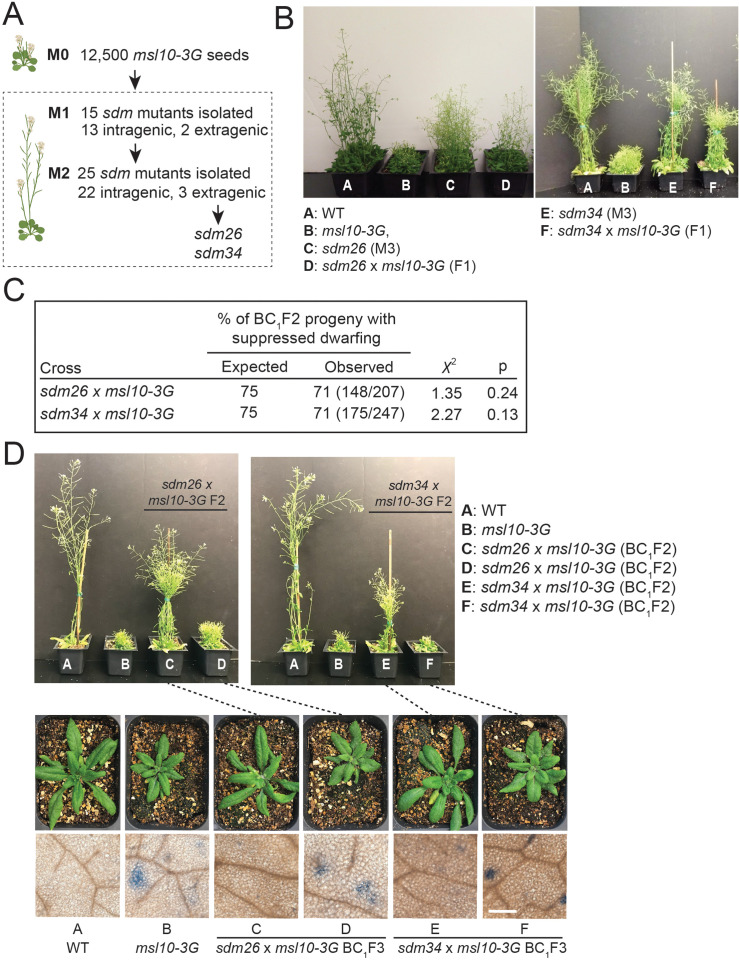
A forward genetic screen identified *sdm26* and *sdm34,* dominant suppressors of *msl10-3G* height and ectopic cell death phenotypes. (**A**) Schematic of the screen. (**B**) Images of the indicated plants after 4–5 weeks of growth. (**C**) Segregation of height phenotypes in the BC_1_F2 generation compared to the expected segregation ratio assuming the *sdm* alleles are dominant. (**D**) Siblings of backcrossed *sdm26* and *sdm34* mutants that were fixed for the *sdm* (suppressed dwarfing) or *msl10-3G* (dwarf) phenotypes. Top: 5-week-old BC_1_F_2_ plants of the indicated genotypes. Middle: 4-week-old BC_1_F_3_ progeny of plants at the top, as indicated with dashed lines. Bottom: leaves of 4-week-old BC_1_F3 plants stained with Trypan blue to assess cell death. These results are representative of at least five other plants for each genotype, in two separate experiments. Scale = 300 µm.

Above, we describe physical interactions between MSL10 and the EPCS components VAP27-1 and VAP27-3, and a functional interaction wherein SYT1 EPCSs were expanded in *msl10-3G* plants. Further evidence for functional interactions between MSL10 and EPCS components came from a genetic screen that was performed at the same time as the above experiments. We used the obvious growth defect of *msl10-3G* plants (Zou et al., 2016; Basu et al., 2020b) as the basis of a visual screen, as illustrated in Figure 5A. EMS-induced suppressor mutants, referred to as *suppressed death from msl10-3G* (*sdm*), were initially isolated based on increased height compared to parental *msl10-3G* plants in the M1 and M2 generations. As *msl10-3G* plants share some of the characteristics of lesionmimic-mutants (Basu et al., 2022), and intragenic mutations are particularly common in suppressor screens of lesionmimic mutants (van Wersch et al., 2016), we sequenced *MSL10* exons in all 40 mutant lines. Indeed, 35 had a missense mutation in the *MSL10* coding or splice-junction sequences (Figure 5—figure supplement 1A). The five remaining *sdm* mutants were presumed to have extragenic suppressor mutations. The mapping-by-sequencing strategy we employed (see below) successfully identified extragenic suppressor mutations for two of these , *sdm26* and *sdm34*.

**Figure 5—figure supplement 1.**
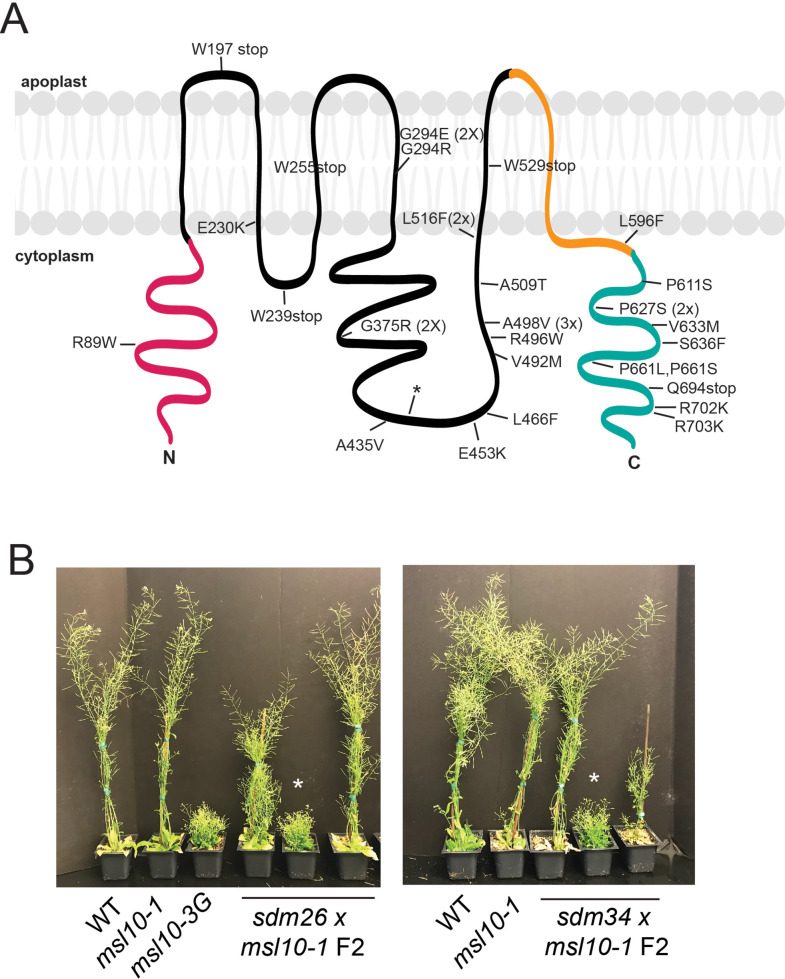
Intragenic *sdm* mutants and tests confirming that *sdm26* and *sdm34* causal mutations are extragenic. (**A**) Intragenic *sdm* mutations mapped onto the predicted MSL10 topology. In pink is the cytosolic N-terminal domain, in teal is the cytoplasmic C-terminal domain, and in orange is the pore-lining MscS domain. The asterisk marks the location of one *sdm* mutation predicted to retain the intron between the first and second exons. (**B**) Pictures of 5-week-old F2 plants. From the *sdm26 × msl10-1* cross, 1 out of 21 F2 plants screened had a dwarf *msl10-3G* phenotype, indicated with the asterisk. From the *sdm34* × *msl10-1* cross, there were 2 out of 23 F2 plants that had the dwarfed phenotype. Other F2 plants from both crosses had either a WT or intermediate height. That the *msl10-3G* (dwarf) phenotype could be recovered after crossing to the null *msl10-1* line indicated that the *sdm26* and *sdm34* alleles were not linked to *MSL10,* confirming that they were extragenic suppressors.

Notably, *sdm26* and *sdm34* mutant plants were taller than *msl10-3G* plants but not as tall as WT plants (Figure 5B). The offspring of both *sdm26* and *sdm34* backcrosses to *msl10-3G* (BC_1_F1 plants) were as tall as their *sdm* parents (Figure 5B). Furthermore, in the BC_1_F2 generation, plants with intermediate height (*sdm* phenotype) were present approximately 3:1 relative to those with the *msl10-3G* dwarf phenotype (Figure 5C), indicating that the *sdm* mutations are dominant in the *msl10-3G* background, at least for this phenotype. When *sdm26* and *sdm34* plants were outcrossed to the *msl10-1* null allele, plants with the parental *msl10-3G* phenotype were recovered in the F2 generation (Figure 5—figure supplement 1B), confirming that the *sdm26* and *sdm34* lesions are extragenic alleles unlinked to *MSL10*. Another characteristic phenotype of *msl10-3G* plants, ectopic cell death, was also suppressed in *sdm26* and *sdm34* leaves compared to those of parental and segregating *msl10-3G* siblings, although the *sdm* mutants exhibited slightly more cell death than WT plants (Figure 5D).

**Figure 6.**
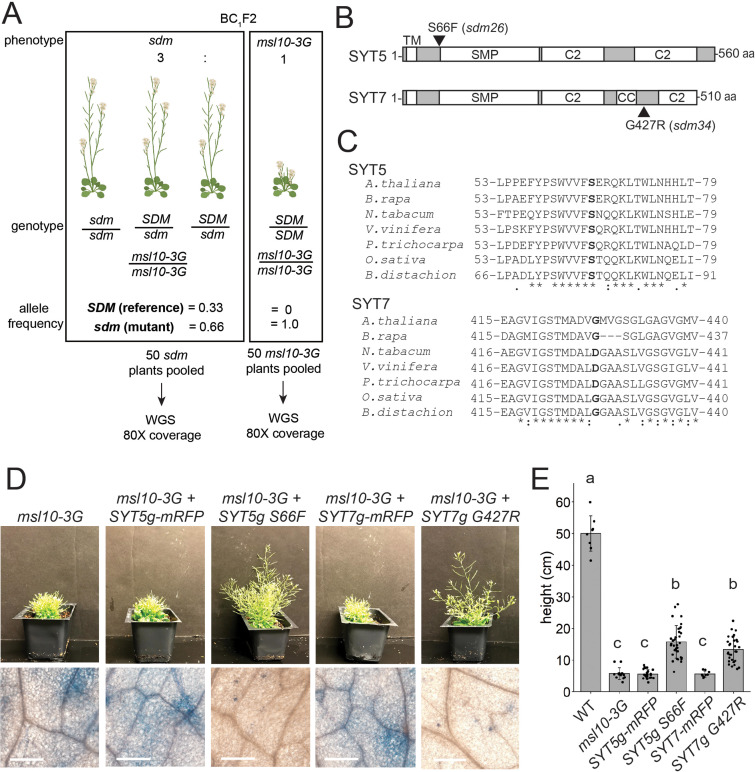
*SYT5 S66F* and *SYT7 G427R* are the causal mutations in *sdm26* and *sdm34*, respectively. (**A**) Overview of backcrossing and mapping-by-sequencing of *sdm* mutants. (**B**) Location of *sdm26* and *sdm34* missense mutations in the SYT5 and SYT7 proteins, respectively. UniProt was used to predict protein domains and their location. TM, transmembrane; SMP, synaptogamin-like mitochondrial-lipid-binding protein domain; CC, coiled coil; C2, Ca^2+^ binding. (**C**) Conservation of Ser66 and Gly427 residues in SYT5 and SYT7 homologs, respectively, in the predicted proteomes of selected angiosperms. (**D, E**) Phenotypes of *msl10-3G* plants expressing WT or *sdm* mutant *SYT5* and *SYT7* transgenes. (**D**) Top: images of representative T1 lines. Bottom: Trypan blue staining of a leaf from the same plants. Scale = 300 µm. (**E**) Mean and standard deviation of plant height of n = 9–32 T1 lines per construct, pooled from two similar experiments. Groups indicated with the same letters are not significantly different as assessed by ANOVA with Scheffe’s post-hoc test.

The whole-genome sequencing strategy we used to identify the mutations responsible for *sdm26* and *sdm34* phenotypes consisted of separating BC_1_F2 plants by phenotype into pools of 50 plants each, extracting genomic DNA from pooled tissue, and sequencing at 80× coverage (Figure 6A). As *sdm26* and *sdm34* are dominant suppressor mutations, we searched for EMS-induced SNPs that (1) had an allele frequency of 0.66 in the pool of plants with the *sdm* phenotype and (2) were absent in the *msl10-3G* phenotype pool. Intervals of adjacent SNPs with such allele frequencies were found on chromosome 1 for *sdm26* (Figure 6—figure supplement 1) and chromosome 3 for *sdm34* (Figure 6—figure supplement 2). We failed to identify clear intervals of linked SNPs with the expected allele frequencies for the other three presumed extragenic mutants.

**Figure 6—figure supplement 1.**
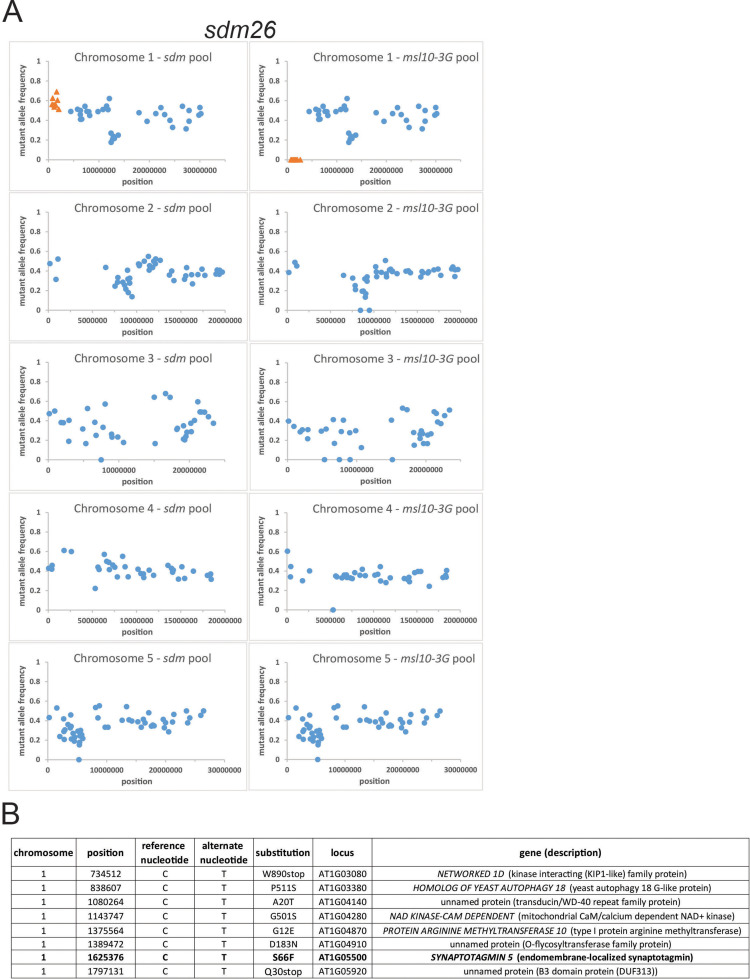
Mapping-by-sequencing reveals the chromosomal regions containing the causal mutations of *sdm26*. *sdm26* was backcrossed to *msl10-3G* plants, and the segregating BC_1_F2 population was pooled by phenotype and sent for whole-genome sequencing (WGS). Segregating BC_1_F2 populations were pooled by phenotype and sent for WGS. (**A**) For each SNP identified, the frequency at which this mutant nucleotide was detected compared to the reference nucleotide was calculated and plotted against its chromosomal position. Regions where SNPs are represented with orange triangles were predicted to contain the causal mutation as mutant alleles were absent in the *msl10-3G* phenotypic pool (dwarfed) and present in the *sdm* phenotypic pool (suppressed dwarfing) near the expected frequency of 0.66. (**B**) Details of the SNPs in the chromosomal intervals identified in (**A**). Gene names and functional descriptions were obtained from TAIR.

**Figure 6—figure supplement 2.**
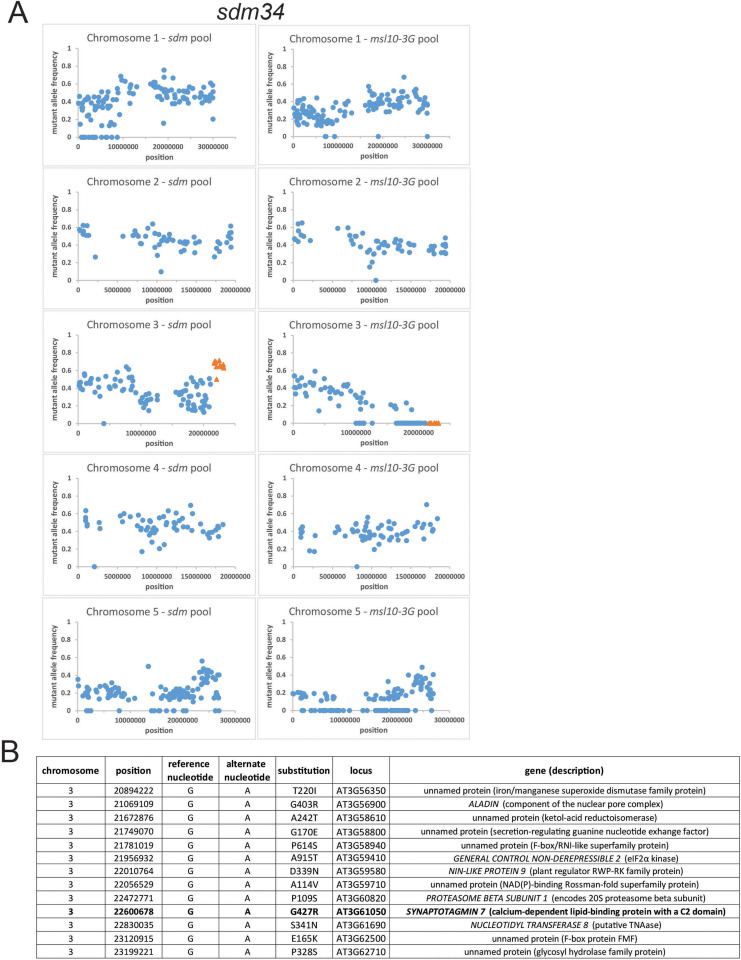
Mapping-by-sequencing reveals the chromosomal regions containing the causal mutations of *sdm34*. *sdm34* was backcrossed to *msl10-3G* plants, and the segregating BC_1_F2 population was pooled by phenotype and sent for whole-genome sequencing (WGS). Segregating BC_1_F2 populations were pooled by phenotype and sent for WGS. For each SNP identified, the frequency at which this mutant nucleotide was detected compared to the reference nucleotide was calculated and plotted against its chromosomal position. Regions where SNPs are represented with orange triangles were predicted to contain the causal mutation as mutant alleles were absent in the *msl10-3G* phenotypic pool (dwarfed) and present in the *sdm* phenotypic pool (suppressed dwarfing) near the expected frequency of 0.66. (**B**) Details of the SNPs in the chromosomal intervals identified in (**A**). Gene names and functional descriptions were obtained from TAIR.

The intervals in *sdm26* and *sdm34* contained 8 and 13 genes, respectively. The *sdm26* genome encoded a missense mutation (Ser66→Phe) in the *synaptotagmin 5* (*SYT5*) gene and the *sdm34* genome encoded a Gly427→Arg substitution in *synaptotagmin 7 (SYT7, CBL1, NTMC2T4;* Figure 6B). SYT5 and SYT7 are known to interact with each other and with SYT1 at EPCSs (Ishikawa et al., 2020; Lee et al., 2020). Given these results, and that MSL10 interacts with EPCS proteins (Figures 1 and 2), the SNPs in *SYT5* and *SYT7* were promising candidates for causing the suppression of the *msl10-3G* phenotypes in *sdm26* and *sdm34*. However, it remained possible that lesions elsewhere in these intervals were instead responsible.

We therefore attempted to recreate the *sdm* phenotypes by expressing *SYT5 S66F* and *SYT7 G427R* from transgenes in unmutagenized *msl10-3G* plants. We expected to see *sdm*-like phenotypes in the T1 generation because the suppressor mutations in *sdm26* and *sdm34* plants were dominant. As anticipated, *msl10-3G+SYT5g S66F* and *msl10-3+SYT7g G427*R T1 plants were taller than untransformed *msl10-3G* plants (Figure 6D). The amount of ectopic cell death was also suppressed compared to *msl10-3G* leaves. WT *SYT5g-mRFP* or WT *SYT7g-mRFP* transgenes had no discernible effect on plant height or ectopic cell death in T1 plants in the *msl10-3G* background. These results provide strong evidence that *SYT5 S66F* and *SYT7 G427R* mutations caused suppression of *msl10-3G* phenotypes in the *sdm26* and *sdm34* mutants, respectively.

To address whether the *sdm26* and *sdm34* mutations might be dominant negative, we crossed *msl10-3G* plants to null *syt5* and *syt7* alleles (Ishikawa et al., 2020). Double *syt5; msl10-3G* and *syt7; msl10-3G* mutants resembled *msl10-3G* plants (Figure 7—figure supplement 1A and B). The inability of null *syt5* and *syt7* alleles to suppress *msl10-3G* phenotypes indicates that the *sdm26 (SYT5 S66F*) and *sdm34 (SYT7 G427R*) alleles do not cause suppression by impairing the function of WT SYT5 or SYT7. Additionally, the null *syt1-2* allele (Ishikawa et al., 2020) had no effect on *msl10-3G* growth defects or ectopic death (Figure 7—figure supplement 1A).

### *sdm26* and *sdm34* alleles do not alter SYT5 or SYT7 localization or MSL10 levels

The SYT5 S66F and SYT7 G427R point mutations occur in different parts of the synaptotagmin proteins and are not located in any of the predicted functional domains (Ishikawa et al., 2020; Lee et al., 2020; UniProt Consortium, 2021; Figure 6B). However, S66 is fully conserved in SYT5 homologs from monocots and dicots and G427 is partially conserved in SYT7 homologs from Brassicacae and monocots (Figure 6C), and thus may be important for structure or function. We first investigated whether the *sdm* point mutations change the localization of SYT5 and SYT7. When transiently expressed in tobacco, SYT5 S66F-mRFP and SYT7 G427R-mRFP had similar localization and dynamics to their WT counterparts, localizing to dynamic ER tubules and to puncta that persisted over time, as previously reported (Ishikawa et al., 2020; Lee et al., 2020; Figure 7—figure supplement 1C; Videos 1–4). Additionally, the *sdm* point mutations did not alter *SYT5* or *SYT7* transcript stability (Figure 7—figure supplement 1D). To rule out a trivial explanation for the suppression of *msl10-3G* phenotypes—that the *sdm26* and *sdm34* alleles decrease MSL10 expression and/or stability—we examined *MSL10p:MSL10-GFP* expression in those backgrounds. We found equivalent MSL10-GFP fluorescence and protein levels in *sdm26* plants compared to their WT siblings, and in *sdm34* plants compared to their WT siblings (Figure 7—figure supplement 1E and F). In summary, the *sdm26* and *sdm34* alleles do not affect MSL10 expression or protein stability, nor SYT5 or SYT7 localization, and must suppress MSL10 signaling in some other way.

**Video 1.** Time-lapse images of SYT5-mRFP in tobacco abaxial leaf epidermal cells. Images were taken every 3 s for 2 min, 5 days post-infiltration.

**Video 2.** Time-lapse images of SYT5 S66F-mRFP in tobacco abaxial leaf epidermal cells. Images were taken every 3 s for 2 min, 5 days post-infiltration.

**Video 3.** Time-lapse images of SYT7-mRFP in tobacco abaxial leaf epidermal cells. Images were taken every 3 s for 2 min, 5 days post-infiltration.

**Video 4.** Time-lapse images of SYT7 G427R-mRFP in tobacco abaxial leaf epidermal cells. Images were taken every 3 s for 2 min, 5 days post-infiltration.

### EPCS expansion is not suppressed in *sdm26* and *sdm34* mutants

**Figure 7.**
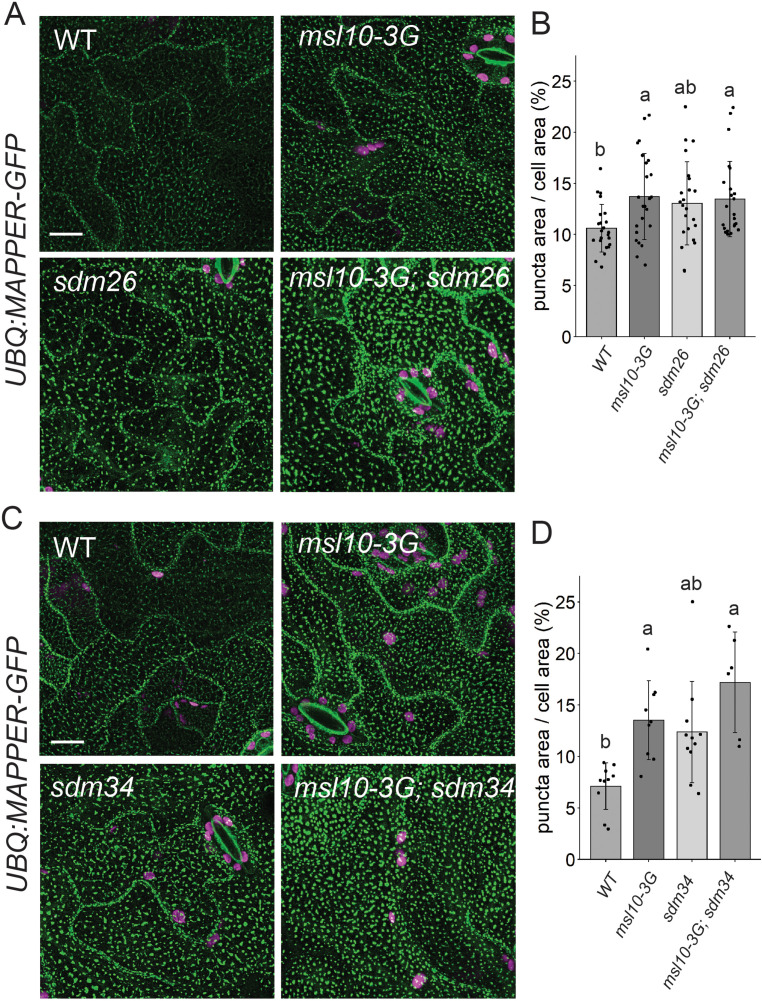
*sdm26* and *sdm34* alleles do not suppress expanded endoplasmic reticulum–plasma membrane contact sites (EPCSs) in *msl10-3G* leaves. (**A, C**) Confocal Z-projections (maximum intensity projection of Z-slices from the top to the middle of cells) of MAPPER-GFP fluorescence in 4-week-old abaxial leaf epidermal cells of the indicated genotypes. Scale = 10 µm. (**B, D**) Quantification of the percentage of the leaf epidermal cell volume taken up by MAPPER-GFP puncta in plants of the indicated genotypes. Each data point represents a biological replicate (the mean value of 20–50 epidermal cells from one plant), n = 6–23 plants per genotype from three separately grown flats. Error bars, SD. Groups indicated with the same letters are not significantly different as assessed by Kruskal–Wallis with Dunn’s post-hoc test when measurements were not normally distributed (**B**) or ANOVA with Scheffe’s post-hoc test when they were (**D**).

Given that SYT1-EPCSs were expanded in *msl10-3G* mutants, we wondered whether increased connections between the ER and PM in *msl10-3G* plants might be responsible for the growth inhibition and ectopic cell death associated with this allele. If this were the case, the enhanced EPCS area observed in *msl10-3G* plants would be suppressed by *sdm26* or *sdm34* alleles. To test this idea, we crossed *UBQ:MAPPER-GFP* plants to the *sdm26* mutant. To our surprise, the larger EPCS area in *msl10-3G* plants (13.7 ± 4.2%) was not suppressed in *sdm26* leaf epidermal cells (13.5 ± 3.7%) (Figure 7A and B). The same observation was made in plants derived from a *UBQ:MAPPER-GFP x sdm34* cross (Figure 7C and D). Thus, differences in ER-PM connectivity, at least as marked by MAPPER-GFP, do not drive the phenotypic differences we observe between WT, *msl10-3G,* and *sdm* plants.

**Figure 7—figure supplement 1.**
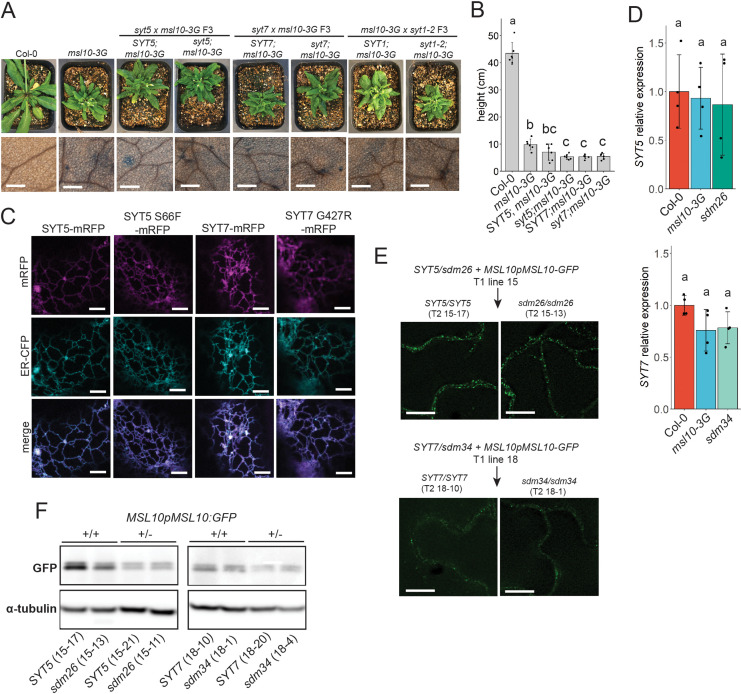
Null *syt1, syt5,* and *syt7* alleles do not suppress *msl10-3G* phenotypes, and *sdm26* and *sdm34* mutations do not alter SYT5 or SYT7 localization, transcript levels, or MSL10 protein levels. (**A**) Plants with the null *syt5, syt7,* or *syt1-2* alleles were crossed to *msl10-3G* plants. Top row: shown are 4-week-old F3 cousins that are homozygous for the *msl10-3G* allele and homozygous for either the *WT* or null alleles. Bottom row: Trypan blue staining of 4-week-old leaves from the same plants. Scale = 300 µm. (**B**) Height of 6-week-old plants homozygous for both the *msl10-3G* allele and either WT or null *SYT5* or *SYT7* alleles. (**C**) Cortical confocal slices of tobacco abaxial epidermal cells transiently co-expressing mRFP-tagged SYT5 and SYT7 constructs under the control of the *UBQ10* promoter and an endoplasmic reticulum (ER) marker (ER-CFP; Nelson et al., 2007). Scale = 5 µm. (**D**) qPCR showing relative *SYT5* and *SYT7* transcript levels in *sdm26* and *sdm34* mutants, normalized to *EF1α* abundance using the 2^-∆∆Ct^ method. RNA was extracted from 4-week-old rosette leaves of backcrossed *sdm26* and *sdm34* mutants. Error bars = SD. Groups indicated with the same letters are not significantly different as assessed by ANOVA with Tukey’s post-hoc test. (**E, F**) *MSL10pMSL10-GFP* transgenes were introduced into plants heterozygous for the *sdm26 (SYT5 S66F*) or *sdm34* (*SYT7 G427R*) alleles (the *msl10-3G* allele had previously been crossed away). Heterozygous T1 plants were identified, and MSL10-GFP stability was compared in T2 siblings that were homozygous for either *SYT* allele. (**E**) Deconvolved images of MSL10-GFP signal in leaf epidermal cells of 4-week-old T2 plants. Scale = 10 µm. (**F**) Immunoblot of MSL10-GFP protein extracted from leaves of 4-week-old T2 siblings. Blots were re-probed with anti-α-tubulin as a loading control. Uncropped images are included as Figure 7—figure supplement 1—source data 1. Figure 7—figure supplement 1—source data 1.Uncropped MSL10-GFP and α-tubulin immunoblots comparing MSL10-GFP expression in plants with and without the SYT5 S66F and SYT7 G427R alleles.

### MSL10 does not interact with SYT5 or SYT7 or reliably influence their localization

**Figure 8.**
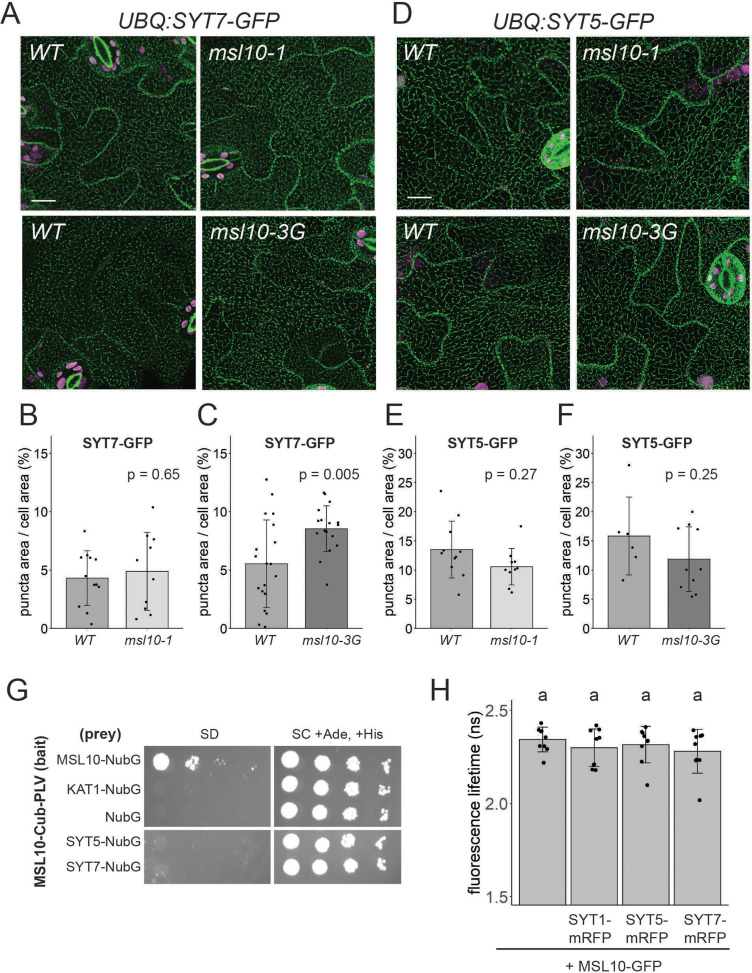
MSL10 does not interact with SYT5 or SYT7 nor reliably alter their localization. (**A, D**) Confocal Z-projections (maximum intensity projection of Z-slices from the top to the middle of cells) of abaxial leaf epidermal cells from 4-week-old plants with the indicated *MSL10* alleles. Scale = 15 µm. Quantification of the percentage of the leaf epidermal cell volume taken up by SYT7-GFP (**B, C**) or SYT5-GFP (**E, F**) puncta in plants in the *msl10-1* or *msl10-3G* backgrounds compared to WT siblings (**A–C**) or cousins (**D–F**). Each data point represents a biological replicate (the mean value of 20–50 epidermal cells from one plant), n = 6–19 plants per genotype from 2 to 4 separately grown flats. Error bars, SD. Means were compared by Student’s *t*-tests. (**G**) Mating-based split-ubiquitin assay testing the interaction of MSL10 with SYT5 and SYT7, performed as in Figure 2A. (**H**) Fluorescence lifetime (τ) of GFP measured using Förster resonance energy transfer-fluorescence lifetime imaging microscopy (FRET-FLIM) when *UBQ:MSL10-GFP* was transiently expressed in tobacco leaves for 5 days, with or without *UBQ:SYT-mRFP* . Each data point represents the value from one field of view (three fields of view per plant from three infiltrated plants for a total of n = 9 for each combination). Error bars, SD. Groups indicated by the same letter are not statistically different according to ANOVA with Tukey’s post-hoc test.

As SYT1-EPCSs were expanded in *msl10-3G* leaf epidermal cells (Figure 4E and G), and SYT1 can interact with SYT5 and SYT7 (Ishikawa et al., 2020; Lee et al., 2020), we asked whether SYT5 and SYT7 localization were also altered in the *msl10-3G* background. We transformed WT Col-0 plants with GFP-tagged constructs under the control of the *UBQ10* promoter and crossed these lines to *msl10-1* and *msl10-3G* plants. Both SYT5-GFP and SYT7-GFP had a partially punctate, partially ER localization, as observed with mRFP-tagged versions expressed transiently in tobacco (Figure 8A and D, Figure 7—figure supplement 1). In some experiments, SYT7-GFP puncta were significantly larger in *msl10-3G* leaf epidermal cells (Figure 8C). However, this observation was not repeatable between experiments (Figure 8—figure supplement 1), suggesting that there are factors other than, or in addition to MSL10 that impact SYT7 EPCS structure. The size of SYT7 EPCSs was unaffected by the *msl10-1* allele, and SYT5-GFP localization was similar in WT, *msl10-3G,* and *msl10-1* leaves.

**Figure 8—figure supplement 1.**
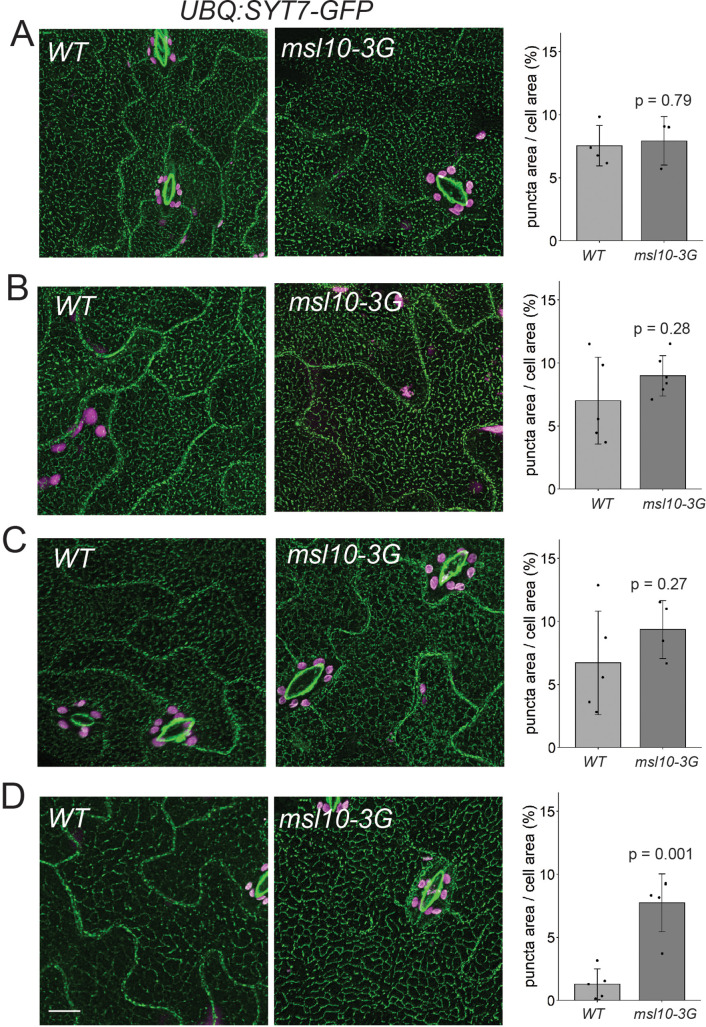
SYT7-GFP localization in leaf epidermal cells varies between experiments. (**A–D**) each represent single experiments of separately grown *UBQ:SYT7-GFP x msl10-3G* F2 siblings. Left: representative deconvolved confocal Z-projections (maximum intensity projection of Z-slices from the top to the middle of cells) of SYT7-GFP fluorescence in 4-week-old abaxial epidermal cells with the indicated genotypes. Right: quantification of the percentage of the leaf epidermal cell volume taken up by SYT7-GFP puncta in plants of each genotype. Each data point represents a biological replicate: the mean value of 10–30 epidermal cells from one plant, 4–6 plants per genotype per experiment. Error bars, SD. Means were compared by Student’s *t*-tests.

We next asked whether MSL10 physically interacts with SYT5 or SYT7. Although SYT5 and SYT7 were not detected in the MSL10 interactome (Figure 1), those experiments were performed in seedlings, whereas the suppression of *msl10-3G* phenotypes by *sdm26* and *sdm34* alleles was observed in adult plants. In the mbSUS assay, yeast expressing SYT5 and SYT7 did not grow on minimal media when mated to yeast expressing MSL10 (Figure 8G). A FRET-FLIM assay also failed to provide evidence for a direct interaction between MSL10 and SYT proteins, as co-expression of mRFP-labeled SYT5, SYT7, and SYT1 did not shift the fluorescence lifetime of MSL10-GFP (Figure 8H). The lack of evidence for physical interactions between MSL10 and SYT1, SYT5, and SYT7 suggests that the observed suppression of the *msl10-3G* phenotype in *sdm26* or *sdm34* mutants is executed indirectly, perhaps through a complex or signaling intermediates.

In summary, in this study we identified three interactions between MSL10 and EPCSss: (1) a physical interaction between MSL10 and VAP27-1 and VAP27-3, (2) a functional interaction in which MSL10 promotes EPCS expansion, and (3) a genetic interaction in which mutations in SYT5 and SYT7 suppress MSL10’s signaling function.

## Discussion

**Figure 9.**
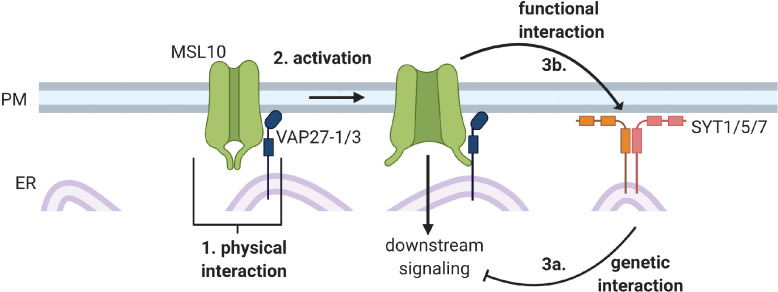
Conceptual model of interactions between MSL10 and EPCS proteins.

The MS ion channel MSL10 has been well studied using electrophysiological approaches (Haswell et al., 2008; Maksaev and Haswell, 2012; Maksaev et al., 2018). Genetic analyses have attributed a variety of roles to MSL10, like the induction of Ca^2+^ transients, reactive oxygen species accumulation, enhanced immune responses, and programmed cell death (Basu and Haswell, 2020a; Moe-Lange et al., 2021; Basu et al., 2022), but we lack a clear understanding of how MSL10 activation leads to these downstream signaling outcomes. Studies using multiple gain-of-function *MSL10* alleles found that MSL10 signaling can trigger cell death independently of ion flux (Veley et al., 2014; Zou et al., 2016; Maksaev et al., 2018; Basu et al., 2020b), though it remains unknown how this occurs. To advance our understanding of the signaling function of MSL10, we used a combination of genetic, proteomic, and cell biological approaches in an attempt to identify MSL10’s signaling partners. We discovered previously unknown interactions between MSL10, which is localized to the plasma membrane, and proteins in the VAP27 and SYT families, which are integral ER membrane proteins. Figure 9 outlines these results and provides a framework for the discussion below. We propose a model wherein (1) a subpopulation of MSL10 directly interacts with VAP27s and creates EPCSs which (2) has implications for MSL10 function and (3) SYTs and MSL10 interact indirectly to modulate MSL10 signaling and SYT1 localization.

### MSL10 physically associates with EPCS proteins

The first indication that MSL10 was part of a protein complex at EPCSs came from our search for proteins that co-immunoprecipitated with MSL10-GFP from seedling microsome extracts. VAP27-1, VAP27-3, and SYT1 were among the most enriched proteins in these pulldowns (Figure 1). Subsequent mbSUS and FRET-FLIM assays support a direct interaction between MSL10 and VAP27-1 and VAP27-3, but not SYT1 or 11 other proteins tested (Figure 2). SYT1, ACT8, and AT3G62360 have been detected in other EPCS proteomes (Ishikawa et al., 2020; Kriechbaumer et al., 2015), and were likely found in the MSL10 interactome because of their proximity to VAP27-1 and VAP27-3. Plant EPCSs typically contain either SYT1 or VAP27-1, but SYT1- and VAP27-1-EPCSs are often found adjacent to each other (Siao et al., 2016), suggesting a physical link between the two types of EPCSs. As MSL10 localizes to the PM (Figure 3; Haswell et al., 2008; Veley et al., 2014), and VAP27-1 and VAP27-3 localize to the ER (Figure 3; Saravanan et al., 2009; Wang et al., 2014), their interaction by definition creates EPCSs. While we cannot exclude the possibility that a small population of MSL10 in another endomembrane compartment interacts with VAP27s, the data presented here support a model wherein a subpopulation of the MSL10 present in the PM interacts with VAPs, thereby forming EPCSs.

### Implications of VAP27-1/3 interaction for MSL10 cell death signaling

The only components of our proteome (among 14 tested proteins) that interacted directly with MSL10 were VAP27-1 and VAP27-3 (Figure 9, point 1). Broadly speaking, VAPs serve to recruit other proteins or protein complexes to the ER membrane. If the client protein is embedded in another organellar membrane, this interaction by definition leads to the formation of a membrane contact site (James and Kehlenbach, 2021). VAP27-1 interacts with SEIPIN2 and SEIPIN3 at ER-lipid droplet contact sites (Greer et al., 2020) and VAP27-3 recruits soluble oxysterol-binding protein-related protein ORP3a to the ER (Saravanan et al., 2009). At EPCSs, *Arabidopsis* VAP27-1 and VAP27-3 interact with clathrin and are required for normal rates of endocytosis, perhaps by recruiting clathrin to the PM (Stefano et al., 2018). Other VAP27-1 interactors include PM intrinsic protein (PIP)2;5, an aquaporin (Fox et al., 2020), AtEH1/Pan1, a protein that recruits endocytic proteins to autophagosomes that form at VAP27-1-containing EPCSs (Wang et al., 2019), and the actin-binding protein NETWORKED 3C (Wang et al., 2014). The cytosolic domains of VAP27-1 and VAP27-3 can interact with phospholipids (Stefano et al., 2018), raising the possibility that they may not need to interact with a protein in another membrane to create a membrane contact site.

Here, we add another VAP27 interactor, one that is associated with mechanical signaling. MSL10 signaling is hypothesized to be activated by membrane tension-induced conformational changes that lead to its dephosphorylation and the activation of its signaling function (Basu et al., 2020b). One could imagine that such post-translational modifications disrupt the ability of MSL10 to interact with VAP27-1 and VAP27-3, thereby activating downstream responses. However, the fact that phosphomimetic (MSL10^7D^), phosphodead (MSL10^7A^), and gain-of-function *msl10-3G* (MSL10 S640L) versions all interacted with VAP27-1 and VAP27-3 (Figure 2—figure supplement 1) implies that MSL10 signaling activation is independent of VAP binding. Rather, MSL10 and VAP27s are likely to interact constitutively, as they did so both in adult leaves, a tissue type in which MSL10-GFP overexpression promotes cell death signaling (Veley et al., 2014) and in seedlings, a stage where MSL10-GFP overexpression has no effect under normal conditions (Basu and Haswell, 2020a).

MSL10 channel and cell death signaling activities are separable (Veley et al., 2014; Maksaev et al., 2018), and VAP27-1 or VAP27-3 could influence either or both of these functions (Figure 9, point 2). In *Zea mays*, interaction with VAP27-1 increases the ability of the PM-localized aquaporin *Zm*PIP2;5 to transport water (Fox et al., 2020). Conversely, the mammalian Kv2.1 K^+^ channel forms non-conducting clusters when it interacts with the VAP27-1 homologs VAPA and VAPB (O’Connell et al., 2010; Fox et al., 2013; Fox et al., 2015; Johnson et al., 2018). It will be interesting to test whether association with VAP27-1 or VAP27-3 alters channel properties of MS such as tension sensitivity. Alternatively, interaction with VAP27s could bring ER-localized regulators of MSL10 signaling into proximity, as is the case for an ER-bound phosphatase and its PM receptor substrate (Haj et al., 2012).

### Point mutations in SYT5 and SYT7 suppress MSL10 signaling

The *msl10-3G* suppressor screen produced two dominant extragenic *sdm* mutants that were successfully mapped to *SYT5* and *SYT7* genes (Figures 5 and 6). Plant synaptotagmins and homologous proteins in mammals (extended-synaptotagmins [E-SYTs]) and yeast (tricalbins) directly bridge the ER and PM via interaction between their C2 domains and PM phospholipids (Schulz and Creutz, 2004; Min et al., 2007; Giordano et al., 2013; Schapire et al., 2008; Pérez-Sancho et al., 2015; Ruiz-Lopez et al., 2021). E-SYTs and tricalbins non-selectively transport glycerolipids between membranes through their synaptotagmin-like mitochondrial lipid-binding (SMP) domains, and *Arabidopsis* SYT1 and SYT3 are hypothesized to transfer diacylglycerol from the PM to the ER during stress conditions (Ruiz-Lopez et al., 2021). The SYT5 S66F mutation (*sdm26* allele) occurs just outside of the predicted SMP domain of SYT5, and the SYT7 G427R mutation (*sdm34* allele) is found between two predicted C2 domains and near a coiled-coil domain (Figure 6). However, both *sdm* alleles were dominant, and both had the same effect of suppressing *msl10-3G* signaling (Figure 9, point 3a). Perhaps these lesions, both of which are in linker regions, influence the large-scale conformational changes that SYTs and E-SYTs are thought to undergo in the presence of Ca^2+^ and certain PM phosphatidylinositol phosphates (Bian et al., 2018; Benavente et al., 2021). This could affect the distance between the ER and PM and the transport of lipids between them, creating a novel lipid environment around MSL10 that might attenuate its ability to activate cell death signaling. Alternatively, the *sdm* mutations in SYT5 and SYT7 might alter the stoichiometry of other proteins at EPCSs, and in turn affect MSL10 function. To test these ideas, lipid transport, phospholipid binding, and interacting proteins should be compared between WT and mutant versions of SYT5 and SYT7.

### SYT1-EPCSs are expanded in *msl10-3G* plants

EPCSs in plant epidermal cells expand in response to environmental perturbations like cold and ionic stress (Lee et al., 2019; Lee et al., 2020; Ruiz-Lopez et al., 2021). We did not find a role for MSL10 in salinity or mannitol-induced EPCS expansion, nor in the shrinking observed after hypo-osmotic shock (Figure 4—figure supplement 1). However, we did find that SYT1 EPCSs were constitutively expanded in leaf epidermal cells of adult *msl10-3G* plants (Figure 4). We did not observe expanded SYT5- or SYT7-EPCSs in *msl10-3G* plants (Figure 8). Although SYT1, SYT5, and SYT7 can interact with each other in immunoprecipitations of whole seedling extracts and in bimolecular fluorescence complementation assays (Ishikawa et al., 2020; Lee et al., 2020), perhaps they are not in a complex together in all cell types or developmental stages as we have drawn in the model for simplicity. We note that it is formally possible that SYT1, SYT5, and SYT7 play nonredundant roles along with MSL10. For example, only *SYT5* and *SYT7* were discovered in the genetic screen, and only SYT1 EPCSs were strongly affected in *msl10-3G* leaves. However, we favor the model that SYT1, SYT5, and SYT7 function redundantly and that each approach described here simply captured the interaction between MSL10 and different individual SYTs depending on their expression in a particular tissue and/or developmental stage.

Why are SYT1-EPCSs expanded in *msl10-3G* leaves? We previously reported that the *msl10-3G* allele promotes a stronger cytosolic Ca^2+^ transient in response to hypo-osmotic cell swelling than is seen in WT seedlings (Basu and Haswell, 2020a). The affinity of SYT1 for PM phospholipids is partially dependent on Ca^2+^ (Schapire et al., 2008; Pérez-Sancho et al., 2015), suggesting that MSL10 could affect SYT1 function. Alternatively, perhaps EPCSs are expanded in *msl10-3G* cells because these cells are already ‘stressed’; *msl10-3G* plants constitutively express markers of wounding and abiotic stress (Zou et al., 2016; Basu et al., 2020b). If overactive stress responses in *msl10-3G* plants increase PM phosphatidylinositol 4,5-bisphosphate (PI(4,5)P_2_) levels, as wounding (Mosblech et al., 2008) or saline conditions (Lee et al., 2019) do, SYT1-EPCS expansion could be promoted. Both of these scenarios are consistent with the fact that we do not observe altered EPCSs in null *msl10-1* leaves. At the moment, the effects we observe on SYT1 area are limited to the gain-of-function *msl10-3G* allele.

However, we did find genetic interactions between the null *msl10-1* allele and a SYT1-GFP overexpression transgene (Figure 4—figure supplement 1). In addition, we were unable to isolate any adult plants overexpressing VAP27-3-GFP in either the null *msl10-1* or gain-of-function *msl10-3G* lines. Taken together, these unexpected genetic results may indicate that the stoichiometry of proteins at plant EPCSs is tightly balanced, and that when disturbed, perturbations of components even in opposing directions can be detrimental. In support of this idea, VAP27-1 gain-of-function and loss-of-function lines both have abnormal root hairs (Wang et al., 2016). Transient overexpression of two EPCS proteins at the same time can drastically alter plant ER and EPCS morphology or even cause necrosis (Wang et al., 2016; Ruiz-Lopez et al., 2021). Additionally, a yeast strain missing all EPCS tethering proteins is viable but cannot tolerate the loss of *OSH4,* a redundant lipid-transport protein (Quon et al., 2018; Quon et al., 2022). Thus, we interpret the synthetic lethality of *MSL10* alleles and VAP27-3 or SYT1 overexpression transgenes as additional evidence that MSL10 functions at plant EPCSs, and we speculate that the ectopic cell death observed in plants overexpressing MSL10-GFP (Veley et al., 2014; Basu et al., 2020b) may be a consequence of altered stoichiometry of EPCS proteins and/or dysfunction of EPCSs. Future studies should examine the dynamics of MSL10, SYTs, and VAP27s in the presence, absence, and overexpression of each other—similar to the study of Siao et al., 2016—to begin to understand the influence they have on each other.

### Implications of having a mechanosensitive ion channel at EPCSs

To our knowledge, MSL10 is the first mechanosensitive ion channel to be found in plant or animal EPCSs, but this may be an unsurprising location to find a mechanosensory protein in any system. It is hypothesized that plant EPCSs interact indirectly with the cell wall (Wang et al., 2017). VAP27-1 and SYT1 are found at Hechtian strands (Wang et al., 2016; Lee et al., 2020), sites of connection between the PM and the cell wall, and the mobility of VAP27-1 is constrained by the presence of a cell wall (Wang et al., 2016). Additionally, plant EPCSs link to the actin and microtubule cytoskeletons (Wang et al., 2014; Zang et al., 2021), which might convey or transduce mechanical information to or from the ER-PM-cell wall interface. By placing the mechanosensitive ion channel MSL10 at EPCSs, our results indicate that EPCSs will be an important nexus for understanding plant mechanotransduction cascades in a cellular context.

## Materials and methods

### Plant lines and growth conditions

All *A. thaliana* lines used in this study are in the Col-0 ecotype. *msl10-3G (rea1*) seeds were derived from an ethyl methanesulfonate (EMS) mutant screen (Zou et al., 2016) and subsequently backcrossed twice (once to parental *RAP2.6::Luc* background and once to Col-0) to remove additional EMS-induced mutations. T-DNA insertion mutants *syt1-2* (SAIL_775_A08), *syt5* (SALK_03961), and *syt7* (SALK_006298) (Ishikawa et al., 2020) and *msl10-1* (Haswell et al., 2008) were obtained from the Arabidopsis Biological Resource Center. *UBQ:MAPPER-GFP* seeds were a gift from Abel Rosado (Lee et al., 2019). Unless otherwise specified, plants were grown on soil at 22°C under a constant light regime (120 µmol m^–2^ s^–1^). To randomize position effects within flats, the position of individual genotypes within flats was changed between replicate experiments.

### Genotyping

DNA was isolated by homogenizing tissue in 300 µL crude extraction buffer (200 mM Tris–HCl pH 7.5, 250 mM NaCl, 250 mM EDTA, and 0.5% sodium dodecyl sulfate) followed by precipitation with an equal volume of isopropanol. Mutant lines were genotyped using the primers indicated in Table 2. The *msl10-3G* point mutation was genotyped using primers 663 and 702 followed by digestion with the *Taq1* restriction enzyme, which cuts only the WT *MSL10* allele. The *sdm26 (SYT5 S66F*) point mutation was genotyped using primers 4155 and 4156 followed by digestion with the *Taq1* restriction enzyme, which cuts the mutant, but not WT *SYT5* sequence. The *sdm34 (SYT7 G427R*) point mutation was genotyped using dCAPs primers 4231 and 4232 and digestion with the *DdeI* enzyme, which cuts the mutant but not the WT *SYT7* allele.

**Table 2.** Primers used in subcloning, genotyping, and sequencing.

#	Name	Sequence (5' → 3')	Purpose
2229	LBb1.3	ATTTTGCCGATTTCGGAAC	Genotyping SALK T-DNA insertion lines
3623	msl10 salk F	GTTGGTTTCTGGGTTTAAGCC	*msl10-1* genotyping
3624	msl10 salk R	TACTTGGAGTAACCGGTGCTG	*msl10-1* genotyping
702	MSL10 exon2 For	GCAACGACTAAGGTTTTGCTG	*msl10-3G* genotyping (for CAPS with Taq1 digestion)
663	MSL10 exon4 Rev	GTTCTTCTTTGTGAGATTAATGTCTTGAGG	*msl10-3G* genotyping (for CAPS with Taq1 digestion), sequencing of *MSL10* genomic DNA
1214	LB1.SAIL	GCTTTTCAGAAATGGATAAATAGCCTTGCTTCC	Genotyping SAIL T-DNA insertion lines
4127	syt1 genotyping F	GAATTGTCCATGTGAAAGTTGTG	*syt1* genotyping
4128	syt5 genotyping F	CTGTCAGCGTTTCTCTTAGAG	*syt5* genotyping
4129	syt5 genotyping R	GAAGAACGTCAACAGTTCAA	*syt5* genotyping
4130	syt7 genotyping F	GAGAAAGCACTAGATAGTTTGACG	*syt7* genotyping
4131	syt7 genotyping R	CTGCTGTTTTGCACCATC	*syt7* genotyping
4055	VAP27-1 For	CACCATGAGTAACATCGATCTGATTG	Amplification of *VAP27-1* ORF for pENTR/D-TOPO cloning
3993	VAP27-1 Rev	TGTCCTCTTCATAATGTATCCC	Amplification of *VAP27-1* ORF for pENTR/D-TOPO cloning
3988	VAP27-3 For	CACCATGAGTAACGAGCTTCTCAC	Amplification of *VAP27-3* ORF for pENTR/D-TOPO cloning
4053	VAP27-3 Rev	TTATGTCCTCTTCATAATGTATCC	Amplification of *VAP27-3* ORF for pENTR/D-TOPO cloning
3990	SYT1 For	CACCATGGGCTTTTTCAGTACGATAC	Amplification of *SYT1* ORF for pENTR/D-TOPO cloning
3991	SYT1 Rev	AGAGGCAGTTCGCCACTC	Amplification of *SYT1* ORF for pENTR/D-TOPO cloning / *syt1* genotyping
4038	ACT8 For	CACCATGGCCGATGCTGATGAC	Amplification of *ACT8* ORF for pENTR/D-TOPO cloning
4039	ACT8 Rev	TTAGAAGCATTTTCTGTGGACAATGA	Amplification of *ACT8* ORF for pENTR/D-TOPO cloning
4024	DL1 For	CACCATGGAAAATCTGATCTCTCTGGT	Amplification of *DL1* ORF for pENTR/D-TOPO cloning
4025	DL1 Rev	CTTGGACCAAGCAACAGC	Amplification of *DL1* ORF for pENTR/D-TOPO cloning
4026	RAB1c For	CACCATGAATCCTGAATATGACTATTTGTT	Amplification of *RAB1c* ORF for pENTR/D-TOPO cloning
4027	RAB1c Rev	TTAAGAGGAGCAGCAGCC	Amplification of *RAB1c* ORF for pENTR/D-TOPO cloning
4020	aCOP1 For	CACCATGTTGACAAAGTTCGAAACC	Amplification of *COPA1* ORF for pENTR/D-TOPO cloning
4052	aCOP1 Rev	CCGGACTTGAGATGGAGAGCATA	Amplification of *COPA1* ORF for pENTR/D-TOPO cloning
4030	LOS1 For	CACCATGGTGAAGTTTACAGCTG	Amplification of *LOS1* ORF for pENTR/D-TOPO cloning
4031	LOS1 Rev	TTAAAGCTTGTCTTCGAAC	Amplification of *LOS1* ORF for pENTR/D-TOPO cloning
4036	MTO3 For	CACCATGGAATCTTTTTTGTTCAC	Amplification of *MTO3* ORF for pENTR/D-TOPO cloning
4037	MTO3 Rev	AGCTTGGACCTTGTTAGAC	Amplification of *MTO3* ORF for pENTR/D-TOPO cloning
3986	AT3G44330 For	CACCATGGCGGAAGAGAAGAAAT	Amplification of *M28 peptidase* ORF for pENTR/D-TOPO cloning
3987	AT3G44330 Rev	TCCCATTTTCACTTTCCG	Amplification of *M28 peptidase* ORF for pENTR/D-TOPO cloning
4032	RPT1a For	CACCATGGTGAGAGATATTGAAGAT	Amplification of *RPT1a* ORF for pENTR/D-TOPO cloning
4033	RPT1a Rev	ATTGTAGACCATATACTTGGG	Amplification of *RPT1a* ORF for pENTR/D-TOPO cloning
4028	CAT2 For	CACCATGGATCCTTACAAGTATCGTC	Amplification of *CAT2* ORF for pENTR/D-TOPO cloning
4029	CAT2 Rev	TTAGATGCTTGGTCTCACG	Amplification of *CAT2* ORF for pENTR/D-TOPO cloning
3994	AT3G62360 For	CACCATGGCGGCCAGTAGGAAG	Amplification of *AT3G44330* ORF for pENTR/D-TOPO cloning
3995	AT3G62360 Rev	GAACGTCTTCTTTCTAGCAACAGC	Amplification of *AT3G44330* ORF for pENTR/D-TOPO cloning
4022	RAN1 For	CACCATGGCTCTACCTAACCAG	Amplification of *RAN1* ORF for pENTR/D-TOPO cloning
4023	RAN1 Rev	CTCAAAGATATCATCATCGTC	Amplification of *RAN1* ORF for pENTR/D-TOPO cloning
3781	MSL10g upstream seq For	CCCACAGTGTTCTTCTATAATC	Amplification of *MSL10* genomic DNA
3782	MSL10g downstream seq Rev	CAGTATCACAACGTTTGGTA	Amplification of *MSL10* genomic DNA
699	MSL10 exon1 For	CAGCACCGGTTACTCCAAGT	Sequencing of *MSL10* genomic DNA
701	MSL10 exon1 For2	ACACATTGGACGAAACAGCA	Sequencing of *MSL10* genomic DNA
1611	MSL10 exon1 Rev	GTTATTGACGTTGAAATTCGCTGCAAGG	Sequencing of *MSL10* genomic DNA
2227	MSL10 exon3 Rev	CGGACTTCTGAAGTAAGCGCTTATCGGTTTCGTGG	Sequencing of *MSL10* genomic DNA
3789	MSL10 intron2 Rev	CCATAATTTATCTTTAAAGAATAAAAGCATG	Sequencing of *MSL10* genomic DNA
4145	SYT5 S66F For	CCTGGGTTGTCTTCTTCGAGCGTCAGAAGTTG	Introducing S66F mutation into *SYT5* by site-directed mutagenesis
4146	SYT5 S66F Rev	CAACTTCTGACGCTCGAAGAAGACAACCCAGG	Introducing S66F mutation into *SYT5* by site-directed mutagenesis
4147	SYT7 G427R For	CAATGGATGCAGTCAGGATGGTGGGAAGTGG	Introducing G427R mutation into *SYT7* by site-directed mutagenesis
4148	SYT7 G427R Rev	CCACTTCCCACCATCCTGACTGCATCCATTG	Introducing G427R mutation into *SYT7* by site-directed mutagenesis
4155	SYT5 For	CACCATGGGTTTCATAGTCGGC	Amplifying *SYT5* for S66F CAPs genotyping/ *SYT5* Gateway cloning
4156	SYT5 internal rev	ACATAAGGCCAGATCTTTGTC	Amplifying *SYT5* for S66F CAPs genotyping/ *SYT5* Gateway cloning
4231	SYT7 dCAPs For	GTAGCACAATGGATGCACTC	Amplifying *SYT7* for G427R dCAPs genotyping
4232	SYT7 internal Rev	ATCCACTACCGACCGCTC	Amplifying *SYT7* for G427R dCAPs genotyping
4157	SYT5 Rev	GGAATCACGATAAATTGATTGA	Amplification of *SYT5* for pENTR/D-TOPO cloning
4158	SYT7 For	CACCATGGGTTTGATTTCTGGG	Amplification of *SYT7* for pENTR/D-TOPO cloning
4159	SYT7 Rev	CTGCTGTTTTGCACCATC	Amplification of *SYT7* for pENTR/D-TOPO cloning
1758	attB1-F	ACAAGTTTGTACAAAAAAGCAGGCTCTCCAACCACCATG	Amplifying genes for split-ubiquitin cloning in yeast
1759	attB2-R	TCCGCCACCACCAACCACTTTGTACAAGAAAGCTGGGTA	Amplifying genes for split-ubiquitin cloning in yeast
3196	EF1α qRT For	ACAGGCGTTCTGGTAAGGAG	Amplifying *EF1*α transcripts for qPCR
3197	EF1α qRT Rev	CCTTCTTCACTGCAGCCTTG	Amplifying *EF1*α transcripts for qPCR
4442	SYT5 qRT For	AGAGGTGAAGCTTGTGCAAG	Amplifying *SYT5* transcripts for qPCR
4443	SYT5 qRT Rev	TGTTGAGTTGACGCGTCTTC	Amplifying *SYT5* transcripts for qPCR
4444	SYT7 qRT For	GCCTTGGACTTGTGAAACTTCC	Amplifying *SYT7* transcripts for qPCR
4445	SYT7 qRT Rev	TCTTCCAACGCAGCCATTTG	Amplifying *SYT7* transcripts for qPCR

### Cloning and generation of transgenic plants

To make *SYT5g S66F* and *SYT7g G427R* constructs, the *SYT5* and *SYT7* genomic sequences were amplified from pGWB553 SYT5g-mRFP and pGWB553 SYT7g-mRFP vectors (Ishikawa et al., 2020), which were a gift from Kazuya Ishikawa, and cloned into the pENTR vector using the pENTR/D-TOPO Cloning Kit (Thermo Fisher). These pENTR constructs were used as templates for site-directed mutagenesis to introduce *SYT5 S66F* or *SYT7 G427R* mutations (primers in Table 2). The mutated genomic sequences were subcloned back into pGWB553 vectors using Gibson Assembly with NEBuilder Hifi DNA Assembly Master Mix (NEB). The WT constructs included a C-terminal mRFP tag (Ishikawa et al., 2020), and the *sdm* constructs had a short, 31aa tag before a stop codon was reached. The resulting constructs were transformed into *msl10-3G* plants using *Agrobacterium tumefaciens* GV3101 and the floral dip method (Clough and Bent, 1998). T1 individuals were identified based on hygromycin resistance.

To make *UBQ:SYT1-GFP, UBQ:SYT5-GFP, UBQ:SYT7-GFP, UBQ:VAP27-1-GFP,* and *UBQ:VAP27-3-GFP* constructs*,* the *SYT1, SYT5, SYT7, VAP27-1,* and *VAP27-3* coding sequences were amplified from Col-0 cDNA using primers in Table 2 and cloned into pENTR using pENTR/D-TOPO, then subcloned into the pUBC-GFP-DEST vector (Grefen et al., 2010) using LR Clonase II (Thermo Fisher recombination). The resulting constructs were introduced into Col-0 plants and transformed individuals were identified based on Basta resistance. T2 plants with moderate GFP fluorescence were crossed to *msl10-1* and *msl10-3G* plants, and homozygous F2 siblings were identified by genotyping and by screening for Basta resistance. To make *UBQ:mRFP-VAP27-1, UBQ:mRFP-VAP27-3, UBQ:SYT1-mRFP, UBQ:SYT5-mRFP, UBQ:SYT7-mRFP,* and *UBQ:MSL10-GFP,* LR Clonase II recombination was used to subclone the coding sequences of *VAP27-1* and *VAP27-3* from pENTR into the pUBN-RFP-DEST vector, *SYT1*, *SYT5*, and *SYT7* into pUBC-RFP-DEST, and *MSL10* into pUBC-GFP-DEST (Grefen et al., 2010).

To make *pK7-mRFP-VAP27-3g*, the *VAP27-3* genomic sequences were amplified from Col-0 genomic DNA. Using Gibson Assembly, this was cloned into the *pK7FWG2* vector backbone, deleting the GFP tag and adding an N-terminal mRFP tag. For co-localization studies, this construct was transformed into Col-0 plants expressing a *MSL10p:MSL10-GFP* transgene (Haswell et al., 2008). T1 plants were identified by kanamycin resistance.

Newly created *Arabidopsis* lines will be submitted to the Arabidopsis Biological Resource Center (abrc.osu.edu), and plasmids deposited to Addgene.

### Microsome isolation and immunoprecipitation

Seeds of Col-0 and *35S:MSL10-GFP* (line 12-3; Veley et al., 2014; Basu et al., 2020b) were densely sown on 1× Murashige and Skoog (MS) plates supplemented with 3% sucrose and grown vertically for 7 days in a 16 hr light/8 hr dark regime. Seedlings (1 g per replicate) were flash-frozen in liquid nitrogen and homogenized to a fine powder using a mortar and pestle. Protein extraction and microsome isolation protocols were modified from Abas and Luschnig, 2010. 1.5 mL of extraction buffer (100 mM Tris–HCl pH 7.5, 25% sucrose, 5% glycerol, 3.3% polyvinylpyrrolidone, 10 mM EDTA, 10 mM EGTA, 5 mM KCl, 1 mM DTT, 0.1 mM PMSF, 2 µM leupeptin, 1 µM pepstatin, 1× plant protease inhibitor cocktail [Sigma P9599], and 1× phosphatase inhibitor cocktails 2 [Sigma P5726] and 3 [Sigma P0044]) was added directly to the mortar and samples were homogenized in buffer for 2 min, then transferred to 1.5 mL tubes and incubated on ice for 10 min. Homogenates were centrifuged at 600 × *g* for 3 min (one replicate) or 10,000 × *g* for 10 min (three replicates) at 4°C to pellet cell debris and organelles. The supernatant was transferred to fresh tubes on ice, and the pellets were resuspended in half of the initial volume of extraction buffer using small plastic pestles. Resuspensions were centrifuged as above. Pooled supernatants were diluted 1:1 with ddH_2_O, then divided among 1.5 mL tubes, each with a maximum volume of 200 µL. Microsomes were pelleted by centrifugation at 21,000 × *g* for 2 hr at 4°C, and the supernatant was discarded.

Microsomal pellets were then resuspended in a total volume of 0.5 mL solubilization buffer (20 mM Tris–HCl pH 7.5, 150 mM NaCl, 2 mM EDTA, 10% glycerol, 0.5% Triton X-100, 0.25% NP-40, 0.1 mM PMSF, 2 µM leupeptin, 1 µM pepstatin, 1× plant protease inhibitor cocktail, and 1× phosphatase inhibitor cocktails 2 and 3) using small plastic pestles. Resuspended microsomes were incubated with end-over-end rotation at 4°C for 1 hr. Meanwhile, 65 µL of GFP-Trap Magnetic Agarose beads (Chromotek) per sample was prepared by washing twice with 1 mL 10 mM Tris–HCl, 150 mM NaCl, 0.5 mM EDTA. To this was added 400 µL of solubilized microsomes and 100 µL of solubilization buffer. Proteins were immunoprecipitated overnight with end-over-end rotation at 4°C. Beads were collected with a magnetic rack, and the flow-through was discarded. Beads were washed three times with 1 mL IP Wash Buffer 1 (20 mM Tris–HCl pH 7.5, 150 mM NaCl, 10% glycerol, 2 mM EDTA, 1% Triton X-100, and 0.5% NP-40), then six times with IP Wash Buffer 2 (20 mM Tris–HCl pH 7.5, 150 mM NaCl, 10% glycerol, 2 mM EDTA), switching to fresh tubes every other wash.

### Liquid chromatography-tandem mass spectrometry (LC-MS/MS)

Proteins were eluted from the GFP-Trap beads by adding 100 µL of 8 M urea, then reduced in 10 mM dithiothreitol for 1 hr at room temperature (RT), and alkylated in the dark (50 mM 2-iodoacetamide) for 1 hr at RT. Excess alkylating agent was quenched with 50 mM DTT for 5 min at RT. Samples were diluted with 900 µL of 25 mM ammonium bicarbonate and digested overnight at 37°C in the presence of 0.35 µg of sequencing grade-modified porcine trypsin (Promega). Peptides were vacuum-dried in a centrifugal evaporator to approximately 250 µL, acidified with 10% trifluoroacetic acid (TFA) (pH < 3), desalted and concentrated on a 100 µL Bond Elut OMIX C18 pipette tip (Agilent Technologies A57003100) according to the manufacturer’s instructions. Peptides were eluted in 50 µL of 75% acetonitrile, 0.1% acetic acid, vacuum-dried in a centrifugal evaporator (Savant Instruments, model number SUC100H), and resuspended in 17 µL 5% acetonitrile, 0.1% formic acid.

Nanoscale liquid chromatography (LC) separation of tryptic peptides was performed on a Dionex Ultimate 3000 Rapid Separation LC system (Thermo Fisher). The protein digests were loaded onto a 20 μL nanoViper sample loop (Thermo Fisher) and separated on a C18 analytical column (Acclaim PepMap RSLC C18 column, 2 μm particle size, 100 Å pore size, 75 µm × 25 cm [Thermo Fisher]) by the application of a linear 2 hr gradient from 4% to 36% acetonitrile in 0.1% formic acid, with a column flow rate set to 250 nL/min. Analysis of the eluted tryptic peptides was performed online using a Q Exactive Plus mass spectrometer (Thermo Scientific) possessing a Nanospray Flex Ion source (Thermo Fisher) fitted with a stainless steel nanobore emitter operated in positive electrospray ionization (ESI) mode at a capillary voltage of 1.9 kV. Data-dependent acquisition of full MS scans within a mass range of 380–1500 m/z at a resolution of 70,000 was performed, with the automatic gain control (AGC) target set to 3.0 × 10^6^, and the maximum fill time set to 200 ms. High-energy collision-induced dissociation (HCD) fragmentation of the top eight most intense peaks was performed with a normalized collision energy of 28, with an intensity threshold of 4.0 × 10^4^ counts and an isolation window of 3.0 m/z, excluding precursors that had an unassigned, +1 or >+7, charge state. MS/MS scans were conducted at a resolution of 17,500, with an AGC target of 2 × 10^5^ and a maximum fill time of 300 ms. Dynamic exclusion was performed with a repeat count of 2 and an exclusion duration of 30 s, while the minimum MS ion count for triggering MS/MS was set to 4 × 10^4^ counts. The resulting MS/MS spectra were analyzed using Proteome Discoverer software (version 2.0.0.802, Thermo Fisher), which was set up to search the *A. thaliana* proteome database, as downloaded from http://www.tair.com/ (TAIR10_pep_20101214). Peptides were assigned using SEQUEST HT (Eng et al., 1994), with search parameters set to assume the digestion enzyme trypsin with a maximum of 1 missed cleavage, a minimum peptide length of 6, precursor mass tolerances of 10 ppm, and fragment mass tolerances of 0.02 Da. Carbamidomethylation of cysteine was specified as a static modification, while oxidation of methionine and N-terminal acetylation were specified as dynamic modifications. The target false discovery rate (FDR) of 0.01 (strict) was used as validation for peptide-spectral matches (PSMs) and peptides. Proteins that contained similar peptides and that could not be differentiated based on the MS/MS analysis alone were grouped to satisfy the principles of parsimony. Label-free quantification as previously described (Silva et al., 2006) was performed in Proteome Discoverer with a minimum Quan value threshold of 0.0001 using unique peptides, and ‘3 Top N’ peptides used for area calculation. All samples were injected in technical duplicate, and the resulting values were averaged. The mass spectrometry proteomics data have been deposited to the ProteomeXchange Consortium via the PRIDE partner repository (Perez-Riverol et al., 2019) with the dataset identifier PXD018747.

Using the Perseus platform (Tyanova et al., 2016), intensity values from mass spectrometry were log_2_ imputed and missing values were replaced with random numbers from a Gaussian distribution with a width of 0.3 and a downshift of 1.8. Statistical significance was determined using *t*-tests. Only proteins with >8 peptide spectrum matches were included in volcano plots.

### mbSUS assay

The coding sequence for the 14 proteins selected from the MSL10 interactome were amplified from Col-0 cDNA using primers in Table 2 and cloned into *pENTR* using pENTR/D-TOPO, then subcloned into the *pK7FWG2* destination vector (Karimi et al., 2002) or BiFC destination vectors (Gehl et al., 2009) using LR Clonase II recombination. These constructs were used as templates for PCR amplification with attB1 For and attB2 Rev primers (Table 2). Following the protocol of Obrdlik et al., 2004 and Basu et al., 2020b, attB-flanked inserts were combined with linearized vectors and transformed into yeast for recombinational in vivo cloning. Inserts were cloned into *pMetYCgate* for a C-terminal fusion with Cub, *pXNgate21-3HA* for a C-terminal fusion with NubG, or *pNXgate33-3HA* for an N-terminal NubG fusion. For integral membrane proteins, split-ubiquitin tags were placed at the terminus predicted to lie in the cytosol. For soluble proteins, the NubG tag was placed on the terminus where fusions had previously reported to be tolerated (or, for unstudied proteins, where homologous proteins had been tagged). NubG vectors and inserts were transformed into THY.AP5 cells (ABRC stock CD3-809, derived from *Saccharomyces cerevisiae* 4932) and selected on synthetic complete (SC) plates lacking tryptophan and uracil. Cub vectors and inserts were transformed into THY.AP4 cells (ABRC stock CD3-808) and selected on SC plates lacking leucine. Transformed cells were mated and diploids selected on SC media lacking tryptophan, uracil, and leucine. Overnight cultures of diploid cells were pelleted, resuspended in dH_2_O to an OD_600_ of 1.0, and 4 µL of a 10× dilution series were spotted onto synthetic minimal (SD) or SC + Ade + His media. Growth was assessed 3 days after plating; growth on SC + Ade + His media tested the presence of both constructs. To quantify the strength of interactions, β-galactosidase activity in liquid cultures was assayed using CPRG as substrate as described in the *Yeast Protocols Handbook* (Takara, PT3024-1).

### FRET-FLIM

*UBQ:mRFP-VAP27-1, UBQ:mRFP-VAP27-3, UBQ:SYT1-mRFP, UBQ:SYT5-mRFP, UBQ:SYT7-mRFP,* and *UBQ:MSL10-GFP* plasmids were transformed into *A. tumefaciens* GV3101. Following the protocol of Waadt and Kudla, 2008, construct pairs were co-infiltrated into *Nicotiana benthamiana* leaves along with *A. tumefaciens* strain AGL-1 (from Herman Scholthof, which harbors p19 to suppress gene silencing). Five days post-infiltration, leaves were imaged using a Leica TCS SP8 Multiphoton microscope fitted with an HC PL IRAPO ×40/1.10 WATER objective. The tunable multiphoton laser was adjusted to its optimum excitation for EGFP (920 nm), and fluorescence lifetimes were recorded in an emission range of 595–570 nm. Using the Leica LASX software’s FLIM tool, an n-Exponential Reconvolution model with one component was used to calculate the average fluorescence lifetime of GFP per image.

### Co-localization analysis

Leaves of plants co-expressing *MSL10p:MSL10-GFP* and *mRFP-VAP27-3g* were imaged using an Olympus FV3000 confocal microscope with a UPLSAPO 100XS oil-immersion objective. Then, 8–12 Z-slices were captured at the equator of abaxial leaf epidermal cells, and these Z-stacks were deconvolved. For each image, regions of interest (ROIs) were defined at the periphery of four different cells. Co-localization was quantified using the ‘Co-localization’ tool of the Olympus cellSens software, using the ‘Rectangle’ mode to automatically estimate thresholds, and the mean of the Mander’s coefficients was calculated from the four ROIs in four Z-slices.

### Confocal microscopy and quantification of ER–PM contact sites

Lines expressing MAPPER-GFP, SYT1-GFP, SYT5-GFP, SYT7-GFP, VAP27-1-GFP, and VAP27-3-GFP under the control of the *UBQ10* promoter were visualized using an Olympus FV3000 confocal microscope with a UPLSAPO 100XS oil-immersion objective. GFP was excited using a 488 nm laser and detected in the 500–540 nm range. Chlorophyll autofluorescence was excited by the same laser and detected in the 650–750 nm range. Z-stacks were taken of abaxial leaf epidermal cells beginning at the top of the cell and ending with an equatorial slice. Z-stacks were deconvolved with the Olympus CellSens software using the Advanced Maximum Likelihood Algorithm with five iterations. The area of MAPPER-GFP or SYT1-GFP puncta was quantified using Fiji (Schindelin et al., 2012). Deconvolved Z-stacks were converted to a Z-projection (sum slices for MAPPER-GFP and maximum intensity for SYT1/5/7-GFP) and the area of each cell was traced and set as an ROI, excluding the periphery of cells where puncta were typically overlapping. After thresholding (between 25–255 for MAPPER-GFP, 100–255 for SYT1-GFP, 85–255 for SYT7-GFP, and 70–255 for SYT5-GFP), the ‘Analyze Particles’ function was used to quantify the percentage of cell area that the puncta represented for each ROI.

### Identification of *suppressed death from msl10-3G* (*sdm*) mutants

250 mg of backcrossed *msl10-3G* seeds (approximately 12,500 seeds) were treated with 0.4% EMS as described in Kim et al., 2006. Mutagenized seeds were sown directly on soil in 40 pools, stratified for 2 days at 4°C, then transferred to a 22°C growth chamber. *sdm* mutants were identified based on increased height compared to parental *msl10-3G* plants 4–5 weeks after sowing, each from individual pools. When multiple plants with *sdm* phenotypes were seen in the same M2 pool, they were assumed to be from the same parent. *sdm* mutants were genotyped to ensure they had the *msl10-3G* point mutation. To see whether *sdm* mutants harbored second-site mutations in the *MSL10* gene, the locus was PCR-amplified using primers 3781 and 3782 and Sanger-sequenced using primers 663, 699, 701, 1611, 2227, and 3789 (Table 2). *sdm26 and sdm34* were backcrossed to *msl10-3G* plants*,* and rosette leaves from 30 to 50 F2 progeny were separated into two pools based on phenotype: *msl10-3G* (dwarfed) or *sdm* (suppressed). Genomic DNA was extracted from pooled tissue following the protocol described in Thole et al., 2014 and submitted to the Genome Technology Access Center at the McDonnell Genome Institute (GTAC@MGI) at the WUSTL Medical Center. Libraries were prepared using the Kapa HyperPrep Kit PCR-free (Roche) and sequenced on an Illumina NovaSeq 6000 S4 Flowcell using 150 nt paired-end reads and 80× coverage. GTAC@MGI aligned reads to the *A. thaliana* Col-0 reference genome (TAIR10.1 assembly), called variants using SAMtools (Li et al., 2009), and annotated them using snpEff (Cingolani et al., 2012). Variants were filtered to include those with a quality score of >20 and a total depth of >5. SNPs that were present in multiple *sdm* mutants were removed, as they were likely present in the parental *msl10-3G* line. For each of the retained SNPs, the allele frequency (mutant/reference) was plotted against chromosomal position.

### Alignment of SYT5 and SYT7 protein sequences

SYT5 and SYT7 homologs in other plant species were identified using the BLAST tools in Phytozome 13 or NCBI using the *Arabidopsis* SYT5 and SYT7 amino acid sequences as queries. To remove sequences that were orthologous to other *Arabidopsis* synaptotagmins, we aligned the obtained sequences to the protein sequences of the seven known synaptotagmins in *Arabidopsis* and constructed a Neighbor-Joining phylogenic tree in Mega 11. We then considered only those sequences that were in the same clade as *At*SYT5 or *At*SYT7 to be SYT5 or SYT7 homologs. SYT5 homologs identified with this method and shown in Figure 6C have the following accession numbers from Phytozome: *B. rapa* B.rapaFPsc v1.3|Brara.J00373.1.p, *V. vinifera* v2.1|VIT_211s0118g00230.2, *P. trichocarpa* v4.1|Potri.018G025000.3.p, *O. sativa* v7.0|LOC_Os04g55220.1, *B. distachyon* v3.2|Bradi5g23880.2.p. From NCBI: *N. tabacum* XP_016446163.1. SYT7 homologs identified in Phytozome include *B. rapa* B.rapaFPsc v1.3|Brara.D00127.1.p, *V. vinifera* v2.1|VIT_215s0048g01410.1, *P. trichocarpa* v4.1|Potri.014G072800.2.p, *O. sativa* v7.0|LOC_Os07g22640.1, *B. distachyon* v3.2|Bradi1g52680.1.p. From NCBI: *N. tabacum* XP_016486625.1.

### Trypan blue staining

A stock solution of Trypan blue (0.025% in a 1:1:1:1 solution of phenol:lactic acid:glycerol:water) was diluted with ethanol to make a working solution (one part Trypan blue:two parts ethanol). This was heated to boiling, then allowed to cool for 10 min. Then, 4- or 5-week-old rosette leaves were submerged in working solution and gently agitated for 15 min. Chlorophyll was removed from leaves by submerging them in an ethanol series, and finally by repeated changes in 1:1:1:1 ethanol:acetic acid:glycerol:water. Leaves were mounted in 20% glycerol for imaging with ×10 magnification.

### Immunoblotting

Rosette leaves were flash-frozen and homogenized in a microcentrifuge tube using a small plastic pestle. Then, 4 µL of 2× sample buffer was added for every 1 mg of tissue, this mixture was denatured for 10 min at 70°C, and cell debris pelleted by centrifugation at 5000 × *g* for 1 min. Supernatants were resolved on 10% SDS-PAGE gels and transferred overnight to PVDF membranes (Bio-Rad) at 100 mA. Blocking and antibody incubations were performed in 5% non-fat dry milk in 1× TBS-T buffer. MSL10 tagged with GFP was detected using an anti-GFP antibody (Takara #632380, RRID:AB_10013427) for 16 hr at a dilution of 1:5000, followed by a 1 hr incubation in HRP-conjugated goat-anti-mouse secondary antibody at a 1:10,000 dilution (Millipore #12-349, RRID:AB_390192). Blots were stripped and reprobed with anti-α-tubulin (Sigma T5168, RRID:AB_477579, 1:30,000 dilution) for 1 hr. Proteins were detected using the SuperSignal West Dura Extended Duration Substrate (Thermo Fisher).

### Gene expression analysis

Rosette leaves were flash-frozen in liquid nitrogen and homogenized into a powder. RNA was extracted using RNeasy Kit (QIAGEN) following the manufacturer’s instructions for plant RNA isolation and on-column DNase digestion. cDNA was synthesized using M-MLV reverse transcriptase (Promega) and oligo(dT) priming. qRT-PCR was performed in technical triplicate using the SYBR Green PCR Master Mix (Thermo Fisher) kit, with primers specific to *SYT5, SYT7,* or *ELONGATION FACTOR 1α (EF1 α*) transcripts (Table 2) on a StepOne Plus Real-time PCR System (Applied Biosystems).

### Accession numbers

The genes utilized in this study have the following *Arabidopsis* Genome Initiative locus codes: *MSL10 (At5G12080), VAP27-1 (At3G60600), VAP27-3 (At2G45140), SYT1 (At2G20990), SYT5 (At1G05500), SYT7 (At3G61050), ACTIN 8 (ACT8, At1G49240), DYNAMIN-LIKE 1 (DL1, At5G42080), RAB GTPase homolog 1C (RAB1c, At4G17530), METHIONINE OVERACCUMULATOR 3 (MTO3, At3G17390), COATOMER ALPHA-1 SUBUNIT (αCOP1, At1G62020),* unnamed protein with a carbohydrate-binding like fold (*At3G62360),* unnamed protein-M28 Zn-peptidase nicastrin (*At3G44330), RAS-RELATED NUCLEAR PROTEIN 1 (RAN1, At5G20010), CATALASE 2 (CAT2, At4G35090), LOW EXPRESSION OF OSMOTICALLY RESPONSIVE GENES 1 (LOS1, At1G56070), REGULATORY PARTICLE TRIPLE-A 1A (RPT1a, At1g53750), POTASSIUM CHANNEL IN ARABIDOPSIS THALIANA 1 (KAT1, At5G46240*).

### Statistical analyses

Statistical analyses were performed in RStudio (v4.1.2), except for chi-squared tests, which were performed in Microsoft Excel. Shapiro–Wilk tests were used to test for normality. The *car* and *agricolae* packages were used to perform ANOVAs and indicated post-hoc tests, and *FSA* and *rcompanion* packages for Kruskal–Wallis and Dunn’s post-hoc tests. Data was visualized using RStudio *ggplot2*, GraphPad Prism 7, and Excel. The Venn diagram shown in Figure 1B was created using http://bioinformatics.psb.ugent.be/webtools/Venn/.

## Data Availability

Mass spectrometry data have been deposited to the ProteomeXchange Consortium via the PRIDE partner repository with the dataset identifier PXD018747, and is included as a Source Data file for Figure 1. The following dataset was generated: McLoughlinF HaswellES 2022Genetic and proteomic screens suggest that signaling by the mechanosensitive ion channel MSL10 is influenced by its association with a synaptotagmin complexPRIDEPXD018747

## Additional files

MDAR checklist
