# Supplementary figures and images for: Unbiased proteomic and forward genetic screens reveal that mechanosensitive ion channel MSL10 functions at ER–plasma membrane contact sites in Arabidopsis thaliana
Source: eLife. 2022 Oct 7;11:e80501. doi: 10.7554/eLife.80501 (PMC9625087; doi:10.7554/eLife.80501)

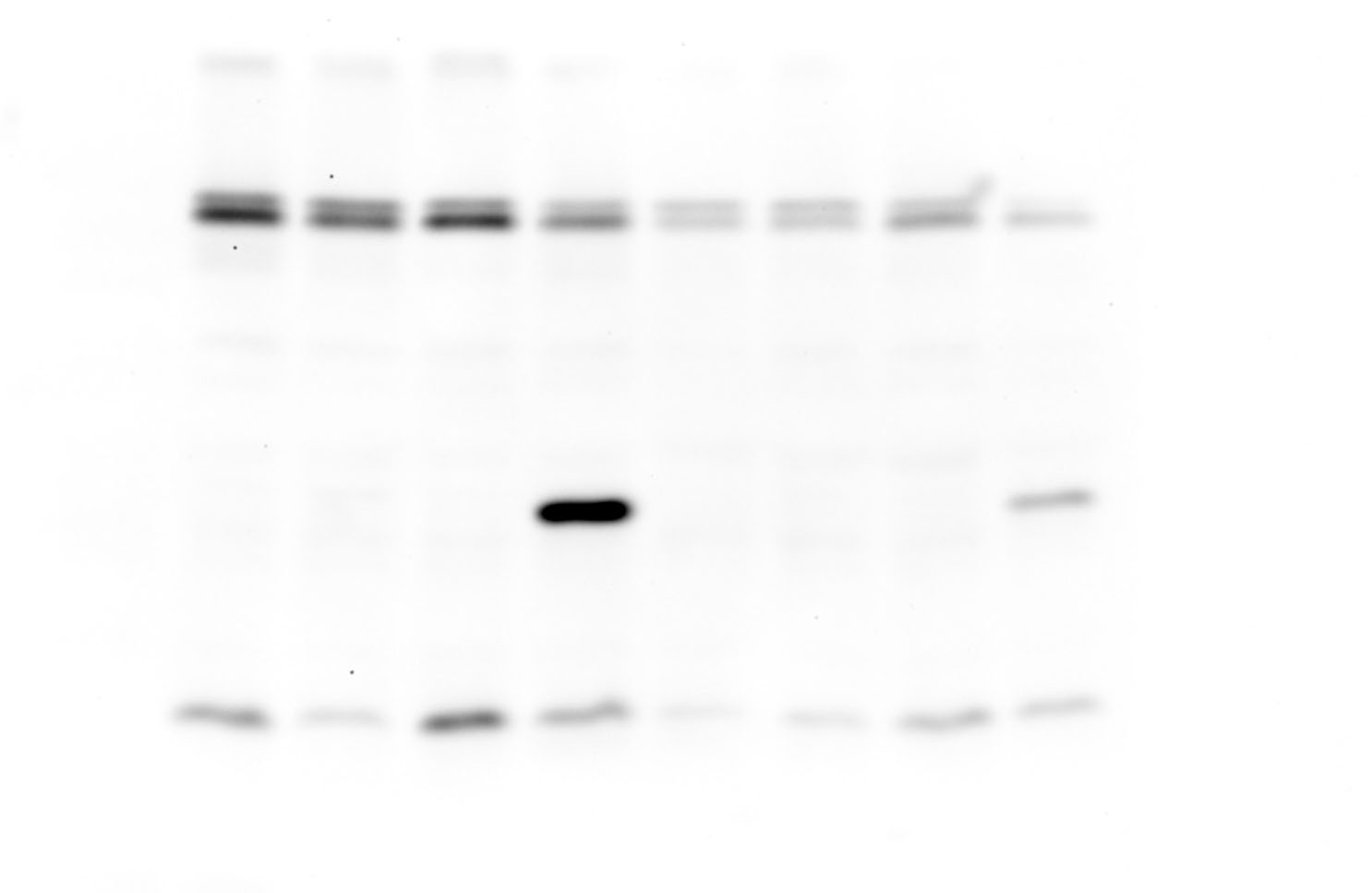

Supplement: Figure 7—figure supplement 1—source data 1. [file elife-80501-fig7-figsupp1-data1.zip › Figure 7 - figure supplement 1 - Source data 1/4-8-22 GFP 10min - SYT5.jpg]

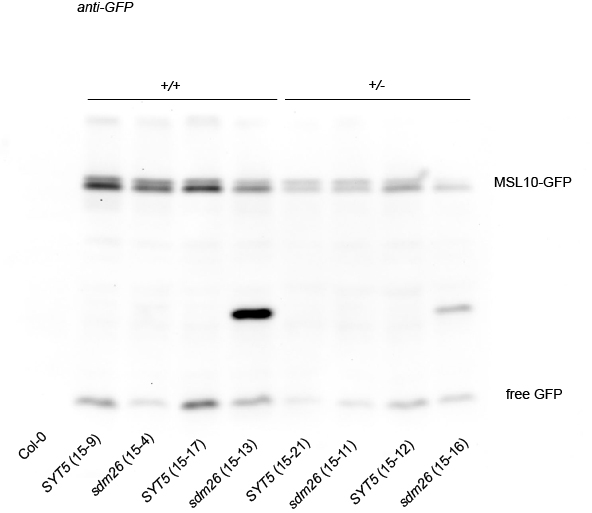

Supplement: Figure 7—figure supplement 1—source data 1. [file elife-80501-fig7-figsupp1-data1.zip › Figure 7 - figure supplement 1 - Source data 1/Figure 7-supplemental figure 1-source data 1.jpg]

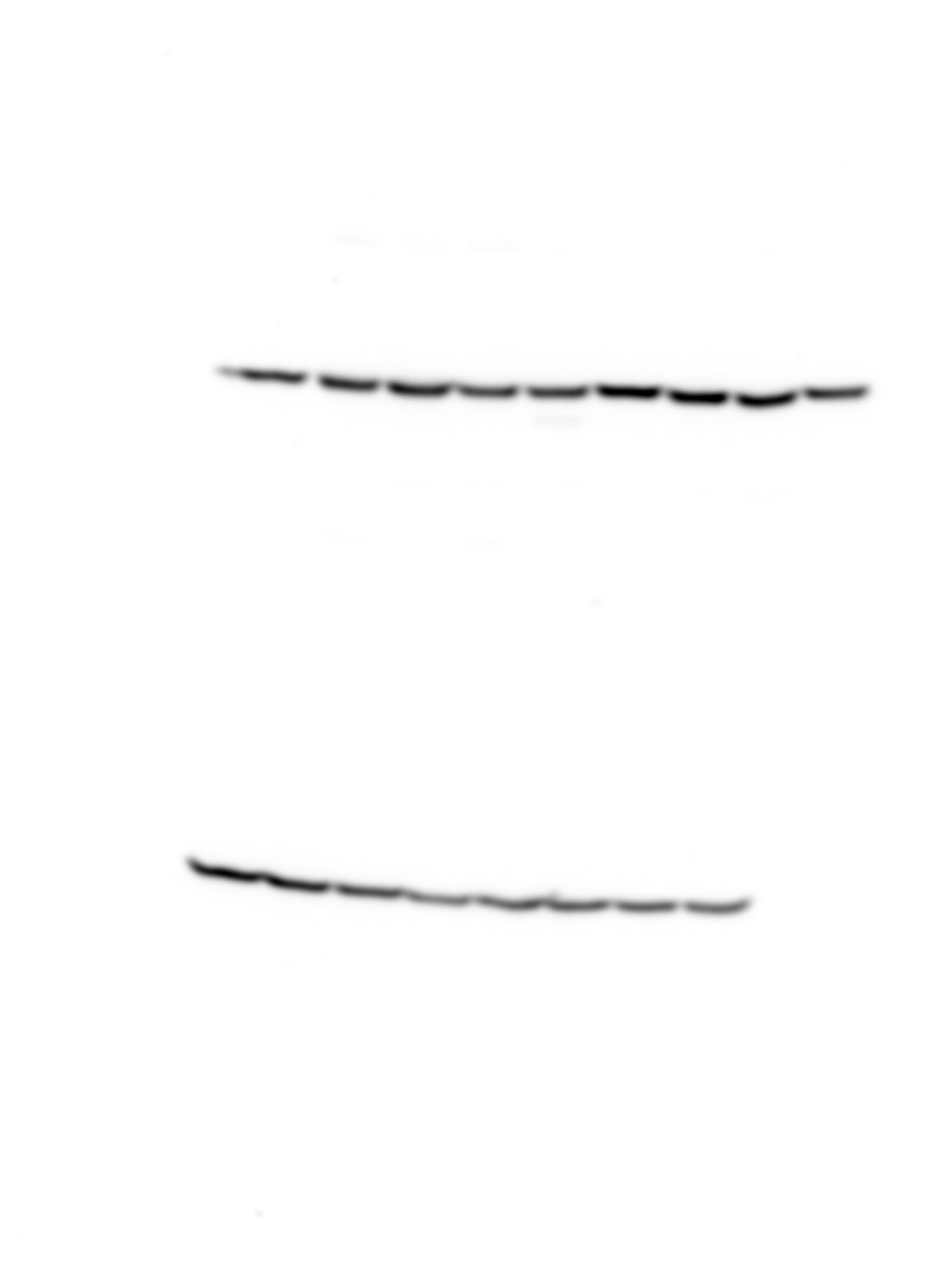

Supplement: Figure 7—figure supplement 1—source data 1. [file elife-80501-fig7-figsupp1-data1.zip › Figure 7 - figure supplement 1 - Source data 1/4-8-22 tubulin 15sec.jpg]

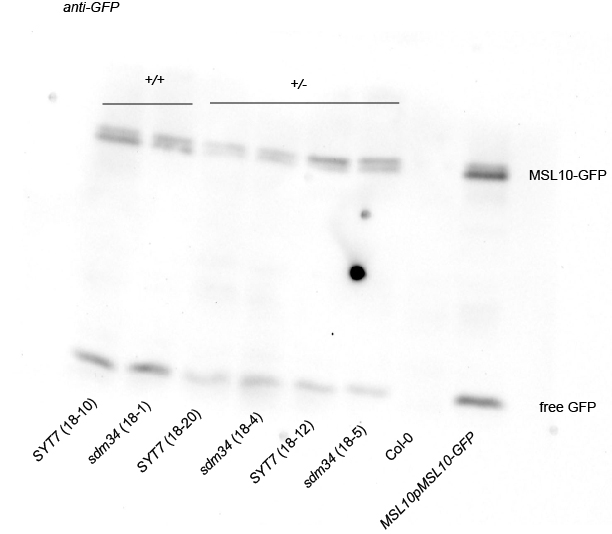

Supplement: Figure 7—figure supplement 1—source data 1. [file elife-80501-fig7-figsupp1-data1.zip › Figure 7 - figure supplement 1 - Source data 1/Figure 7-supplemental figure 1-source data 2.jpg]

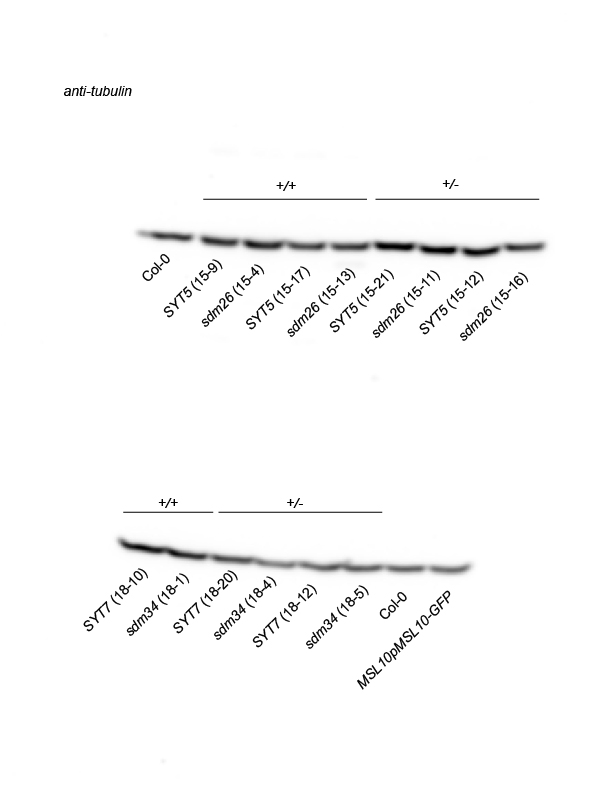

Supplement: Figure 7—figure supplement 1—source data 1. [file elife-80501-fig7-figsupp1-data1.zip › Figure 7 - figure supplement 1 - Source data 1/Figure 7-supplemental figure 1-source data 3.jpg]

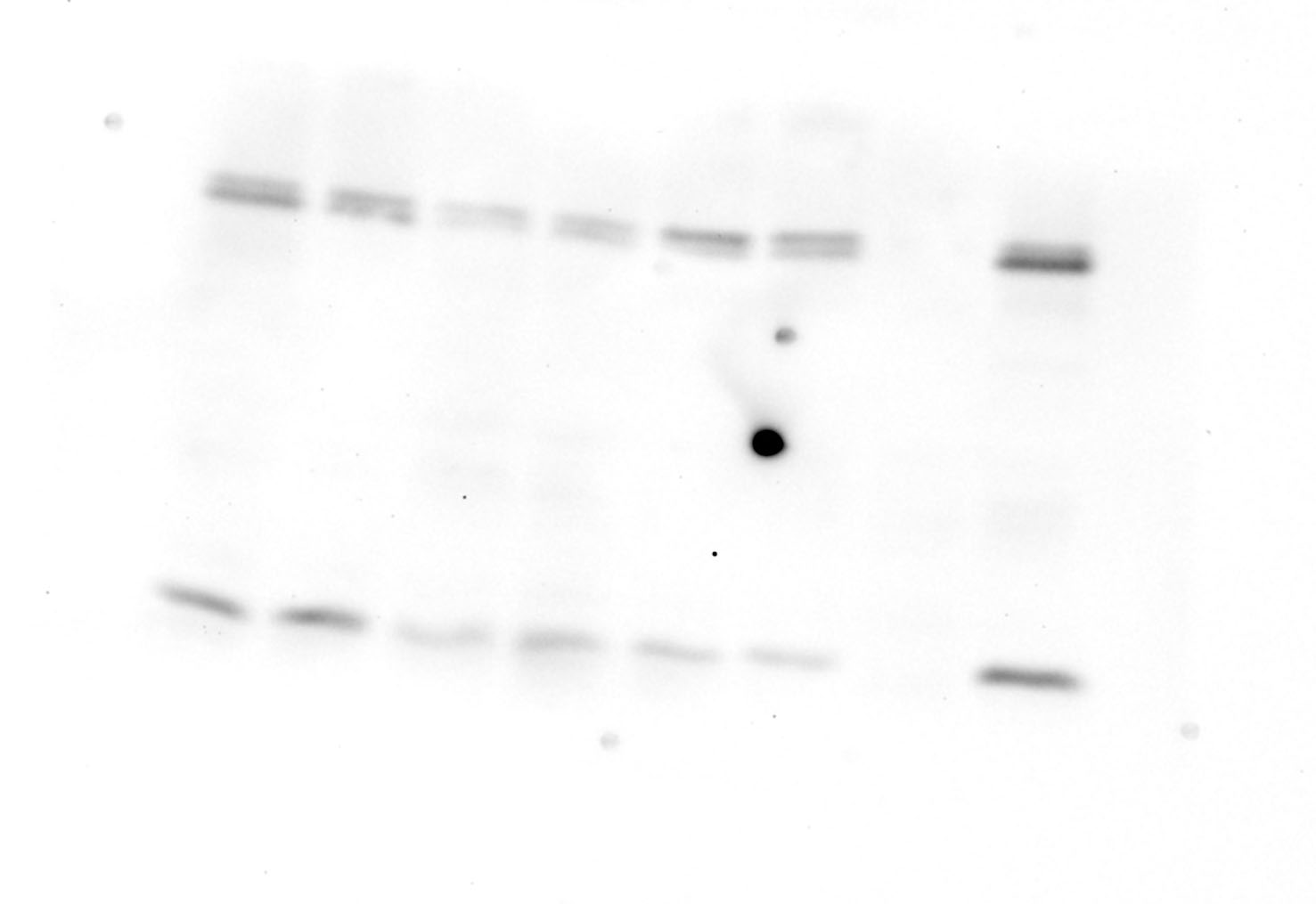

Supplement: Figure 7—figure supplement 1—source data 1. [file elife-80501-fig7-figsupp1-data1.zip › Figure 7 - figure supplement 1 - Source data 1/4-8-22 GFP 10min - SYT7.jpg]
