# Supplementary material for: Unbiased proteomic and forward genetic screens reveal that mechanosensitive ion channel MSL10 functions at ER–plasma membrane contact sites in Arabidopsis thaliana
Source: eLife. 2022 Oct 7;11:e80501. doi: 10.7554/eLife.80501 (PMC9625087; doi:10.7554/eLife.80501)
Supplement: Peer review history

# Editor report: Editor's evaluation

**DOI:** 10.7554/eLife.80501.sa0

**Authors:** Yoselin Benitez-Alfonso

**Affiliations:** https://ror.org/024mrxd33 University of Leeds United Kingdom

The work offers new avenues to investigate the role of mechanosensitive channels in plant development and specifically the mechanism underlying their signaling function. Congratulations on your contributions to this emerging and exciting area of research. The results reported here prepare the grounds to further work aiming to identify how these channels integrate with VAPs and SYTs, how MSL10 contribute to EPCS expansion, and how they function to determine plant growth and responses to the environment.

# Decision letter

**DOI:** 10.7554/eLife.80501.sa1

**Affiliations:** https://ror.org/024mrxd33 University of Leeds United Kingdom; Wageningen University Netherlands; https://ror.org/04v2twj65 Oxford Brookes University United Kingdom

Our editorial process produces two outputs: (i) public reviews designed to be posted alongside the preprint for the benefit of readers; (ii) feedback on the manuscript for the authors, including requests for revisions, shown below. We also include an acceptance summary that explains what the editors found interesting or important about the work.

**Decision letter after peer review:**

Thank you for submitting your article "Unbiased proteomic and forward genetic screens reveal that mechanosensitive ion channel MSL10 functions at ER-plasma membrane contact sites in *Arabidopsis thaliana*" for consideration by *eLife*. Your article has been reviewed by 2 peer reviewers, and the evaluation has been overseen by a Reviewing Editor and Jürgen Kleine-Vehn as the Senior Editor. The following individuals involved in the review of your submission have agreed to reveal their identity: Dolf Weijers (Reviewer #1); Verena Kriechbaumer (Reviewer #2).

The reviewers have discussed their reviews with one another, and the Reviewing Editor has drafted this to help you prepare a revised submission.

Essential revisions:

Both reviewers indicated that the study is novel and interesting. Although there is a large amount of data approaching the research question from various angles and applying different methodologies, both reviewers agreed that there is no clear evidence of a mechanism of action and that the model proposed is largely hypothetical.

The reviewers propose a few experiments be completed before resubmission to strengthen the results and support the conclusions. The following points must be addressed:

1) Better resolution, more pictures to demonstrate that MSL10 resides only in the plasma membrane and/or in the EPCS, clearer localizations of MAPPER, SYT1/5/7, VAP27, and colocalization of MSL10 with VAP27 using controls and analysed in cortical images.

2) functional analysis/ characterization of the MSL10 overexpressor and tagged line which was used to generate the proteomics. Other controls are suggested by reviewer 2.

3) Ideally, the authors will be able to generate and functionally analyze an MSL10 variant that does not localize to EPCSs or that can no longer interact with VAP27 or SYT1 (see reviewer 1). This data is essential to support the mechanism of action.

We hope you can provide the revisions proposed and look very much forward to reading your revised manuscript.

*Reviewer #1 (Recommendations for the authors):*

A few suggestions for improvement:

1. Throughout the manuscript, and particularly in the model in Figure 8, MSL10 is proposed to reside only in the plasma membrane. How well has this been established? Quite a bit of the interpretation of the manuscript's data rests on this. It would be important to clearly show this.

2. In the same vein, all localizations in the manuscript (MAPPER, SYT1/5/7, VAP27) are shown in small details of individual leaf epidermal cells. This makes it impossible to evaluate whether there are other cellular pools that may contribute to interaction and function.

3. The proteomics is performed on a line that overexpresses a tagged version of MSL10. Data showing the faithful behavior of tagged and overexpressed MSL10 protein are required to take the MS data at face value.

4. The colocalization of MSL10 with VAP27 in Figure 2D/E is difficult to interpret without an unrelated protein as a control. Is the correlation coefficient low or high? The fluorescence images themselves show very little colocalization.

5. While the localization of various markers is analysed in the msl10 mutant alleles, it appears that these are all static analyses, while EPCSs are not likely to be static. Did the authors address the dynamics of these markers in mutant backgrounds?

6. While the data strongly suggest a role for MSL10 at EPCSs, this is not directly demonstrated, nor is the relevance of protein-protein interactions for MSL10 function shown. While this may be challenging, any analysis of the function of an MSL10 variant that does not localize to EPCSs or that can no longer interact with VAP27 or SYT1 would be very helpful in inferring such relevance.

*Reviewer #2 (Recommendations for the authors):*

I very much appreciate the variety of approaches and methods and the description of rather interesting data.

Some comments for improvement of the manuscript:

1) The abstract could be more informative and detailed about the results presented in the work. Here I also would avoid "MSL10 can be found at EPSC" as I don't think this is really shown in the manuscript but rather indirect by interaction with VAP/SYT.

2) line 98: instead of WT (Col0) seedlings, should it be "microsomes"?

3) In immunoprecipitation, a control using an unrelated/truncated plasma membrane protein and a GFP control would be advantageous over just a WT control. It would also be good to add the truncated list of 239 as a separate excel tab to source data 1.

4) In the further analysis and comparison RTN3 and 6 are included as EPCS proteins for which I don't think there is any evidence. They might even obstruct the comparative approach here. Why are the VAPs not included in figure 1B?

5) The co-localisation of MLS10 and VAP27 (Figure 2D) should be analysed in cortical images.

6) In figure 3, the order of images does not correspond to the order of images called out in the text (g and d should be swopped). Adding text ("mapper" and "SYT") to B/C and E/F also would speed up the understanding of this figure to the reader. This is of course in the figure legend but this is quite extensive and it would be helpful in the figure as well.

7) A very brief summary of the data presented at the end of the results is always appreciated especially with an extensive dataset.

# Reply: Author response

**DOI:** 10.7554/eLife.80501.sa2

Essential revisions:

1) Better resolution, more pictures to demonstrate that MSL10 resides only in the plasma membrane and/or in the EPCS, clearer localizations of MAPPER, SYT1/5/7, VAP27, and colocalization of MSL10 with VAP27 using controls and analysed in cortical images.

We made several changes to the figures to address this suggestion:

1) To demonstrate that MSL10 resides only in the plasma membrane and/or in the EPCS: We have previously shown that MSL10-GFP localizes to the PM of root cells when expressed at native levels (Haswell et al., 2008). Previous publications detected MSL10 channel activity in the PM of root (Haswell et al., 2008) and callus (Tran et al., 2020) protoplasts. To better establish MSL10-GFP localization when transiently expressed in tobacco epidermal cells, we have moved panels 2D and 2E to a new figure 3 and added cortical images of VAP27/MSL10 co-localization in tobacco to Figure 3c. New equatorial images of the same cells in Figure 3d show clearly that MSL10 resides in the PM and VAP27-1/3 in the ER; their interaction should, by definition, create EPCSs. We now address this more explicitly in the discussion and add a caveat:

“As MSL10 localizes to the PM (Figure 3; Haswell et al., 2008; Veley et al., 2014), and VAP27-1 and VAP27-3 to the ER (Figure 3; Saravanan et al., 2009; Wang et al., 2014), their interaction is predicted by definition to create EPCSs. While we cannot exclude the possibility that a small population of MSL10 in another endomembrane compartment interacts with VAP27s, the data presented here support a model wherein a fraction of the MSL10 present in the PM interacts with VAPs, thereby forming EPCSs.”

2) For localization of MAPPER, SYT1/5/7, and VAP27-1 shown in Figures 3, 6, and 7: All these images are projections of Z-stacks taken from the top of the epidermal cells to their equator; thus, they represent the entire cellular pools of these proteins. We have added text to the figure legends to clarify this point:

“Confocal Z-projections (maximum intensity projection of Z-slices from the top to the middle of cells) of abaxial leaf epidermal cells…”

In addition, we have replaced the images in Figures 4, 7, and 8 (previously Figures 3, 6, and 7) with zoomed-out versions that show more epidermal cell surface area.

3) For co-localization of MSL10 with VAP27, we have added cortical and equatorial images of these proteins co-expressed in tobacco leaves to Figure 3c and 3d. As discussed below, we have added text to clarify our interpretation that only a subpopulation of MSL10 co-localizes with VAP27s. Because we do not claim that most of MSL10 co-localizes with VAP27s, we do not think the addition of unrelated controls for co-localization would significantly alter this conclusion.

2) Functional analysis/ characterization of the MSL10 overexpressor and tagged line which was used to generate the proteomics. Other controls are suggested by reviewer 2.

MSL10-GFP overexpression lines have been characterized in previous publications from our group. In (Maksaev and Haswell, PNAS, 2012) we showed that adding a C-terminal GFP-tag to MSL10 does not alter its electrophysiological properties when expressed in *Xenopus oocytes*. In Veley et al., 2014, we characterized the phenotypes of plants overexpressing MSL10-GFP and interpreted them as gain-of-function. In (Basu, et al., J Ex Bot, 2020), (Basu and Haswell, Current Biology 2020), and (Basu et al., Mol Plant Micro Interact, 2021) we showed that overexpression of MSL10-GFP has the same phenotypic effects as other gain-of-function alleles, including the one used for the genetic screen in this paper (msl10-3G). These phenotypes include dwarfing, ectopic cell death, elevated ROS production, altered gene expression, the induction of programmed cell death, and resistance to pathogens. We regret that these points were not made clear in the first version of the manuscript. We added a sentence to the introduction and to the results that establish the relationship between MSL10-GFP overexpression, the msl10-3G allele, and normal MSL10 function:

In the introduction: “MSL10 gain-of-function lines –including MSL10-GFP overexpressors (Veley et al., 2014) and the EMS-induced point mutant msl10-3G (Zou et al., 2016)–lead to constitutive growth retardation and ectopic cell death (Basu et al., 2020).”

In the results: “GFP-tagged MSL10, which has the same electrophysiological and cell death signaling properties as untagged MSL10 (Maksaev et al., 2012; Basu et al., 2020), was used as bait for immunoprecipitation-mass spectrometry.”

3) Ideally, the authors will be able to generate and functionally analyze an MSL10 variant that does not localize to EPCSs or that can no longer interact with VAP27 or SYT1 (see reviewer 1). This data is essential to support the mechanism of action.

We agree that this would be an excellent experiment. Unfortunately, such a variant does not exist, and all our efforts to create one have so far failed. For example, preliminary data suggest that the N-terminus of MSL10 is required for its interaction with VAP27 in the yeast two-hybrid (data not shown), but this domain is required for normal trafficking in plant cells, so we were unable to validate this in planta. We hope that the reviewers and editors will appreciate the leap forward in understanding MSL10 function provided by the current manuscript.

We hope you can provide the revisions proposed and look very much forward to reading your revised manuscript.

Reviewer #1 (Recommendations for the authors):

A few suggestions for improvement:

1. Throughout the manuscript, and particularly in the model in Figure 8, MSL10 is proposed to reside only in the plasma membrane. How well has this been established? Quite a bit of the interpretation of the manuscript's data rests on this. It would be important to clearly show this.

We have previously shown that MSL10-GFP localizes to the PM of root cells when expressed at native levels (Haswell et al., 2008). Previous publications detected MSL10 channel activity in the PM of root (Haswell et al., 2008) and callus (Tran et al., 2020) protoplasts. To better establish MSL10-GFP localization when transiently expressed in tobacco epidermal cells, we have added cortical images of VAP27/MSL10 co-localization in tobacco to Figure 3. Equatorial images of the same cells show clearly that MSL10 resides in the PM and VAP27-1/3 in the ER; their interaction should, by definition, create EPCSs. We now address this more explicitly in the discussion and add a caveat:

“As MSL10 localizes to the PM (Figure 3; Haswell et al., 2008; Veley et al., 2014), and VAP27-1 and VAP27-3 to the ER (Figure 3; Saravanan et al., 2009; Wang et al., 2014), their interaction is predicted by definition to create EPCSs. While we cannot exclude the possibility that a small population of MSL10 in another endomembrane compartment interacts with VAP27s, the data presented here support a model wherein a fraction of the MSL10 present in the PM interacts with VAPs, thereby forming EPCSs.”

2. In the same vein, all localizations in the manuscript (MAPPER, SYT1/5/7, VAP27) are shown in small details of individual leaf epidermal cells. This makes it impossible to evaluate whether there are other cellular pools that may contribute to interaction and function.

All these images are projections of Z-stacks taken from the top of the epidermal cells to their equator; thus, they represent the entire cellular pools of these proteins. We have added text to the figure legends to clarify this point:

“Confocal Z-projections (maximum intensity projection of Z-slices from the top to the middle of cells) of abaxial leaf epidermal cells…”

In addition, we have replaced the images in Figures 3, 6, and 7 (now Figures 4, 7, and 8) with zoomed-out versions that show more epidermal cell surface area.

3. The proteomics is performed on a line that overexpresses a tagged version of MSL10. Data showing the faithful behavior of tagged and overexpressed MSL10 protein are required to take the MS data at face value.

MSL10-GFP overexpression lines have been characterized in previous publications from our group. In (Maksaev and Haswell, PNAS, 2012) we showed that adding a C-terminal GFP-tag to MSL10 does not alter its electrophysiological properties when expressed in *Xenopus oocytes*. In Veley et al., 2014, we characterized the phenotypes of plants overexpressing MSL10-GFP and interpreted them as gain-of-function. In (Basu, et al., J Ex Bot, 2020), (Basu and Haswell, Current Biology 2020), and (Basu et al., Mol Plant Micro Interact, 2021) we showed that overexpression of MSL10-GFP has the same phenotypic effects as other gain-of-function alleles, including the one used for the genetic screen in this paper (msl10-3G). These phenotypes include dwarfing, ectopic cell death, elevated ROS production, altered gene expression, the induction of programmed cell death, and resistance to pathogens. We regret that these points were not made clear in the first version of the manuscript. We have added a sentence to the introduction and to the results that establish the relationship between MSL10-GFP overexpression, the msl10-3G allele, and normal MSL10 function:

In the introduction: “MSL10 gain-of-function lines –including MSL10-GFP overexpressors (Veley et al., 2014) and the EMS-induced point mutant msl10-3G (Zou et al., 2016)–lead to constitutive growth retardation and ectopic cell death (Basu et al., 2020).”

In the results: “GFP-tagged MSL10, which has the same electrophysiological and cell death signaling properties as untagged MSL10 (Maksaev et al., 2012; Basu et al., 2020), was used as bait for immunoprecipitation-mass spectrometry.”

4. The colocalization of MSL10 with VAP27 in Figure 2D/E is difficult to interpret without an unrelated protein as a control. Is the correlation coefficient low or high? The fluorescence images themselves show very little colocalization.

We agree that the degree of colocalization is low, but do not consider this a concern. Low co-localization between a VAP and a PM-localized protein, despite a direct protein-protein interaction, has previously been observed with Zea mays VAP27-1 and a PM-localized aquaporin (Fox et al., 2020). Their interpretation is the same as ours—that only a subpopulation of integral PM proteins interacts with VAP27s—a point we neglected to make in the first draft of the manuscript. We have added text to make this clear:

“Due to low endogenous expression of MSL10-GFP and cell wall autofluorescence, we could not obtain a cortical image of MSL10-GFP and mRFP-VAP27-3 co-localization in Arabidopsis. We could however examine co-localization in cortical and equatorial slices of tobacco leaf epidermal cells transiently overexpressing MSL10-GFP and mRFP-VAP27-3 or mRFP-VAP27-1 (Figure 3c-d). These images confirm what we observed in Arabidopsis—that only a small subpopulation of MSL10 co-localized with VAP27s, and vice versa. This is similar to what has been observed with the PM-localized aquaporin ZmPIP2;5 and ZmVAP27-1 (Fox et al., 2020). Additionally, they highlight that the majority of MSL10-GFP, even when overexpressed, traffics to the plasma membrane whereas mRFP-VAP27-1 and mRFP-VAP27-3 are found in the ER just below.”

We do not think that including an unrelated protein as a control would significantly alter the conclusions that (1) MSL10 and VAP27s can be found at the same subcellular locations to allow for an interaction and (2) that only a subpopulation of the proteins interact with each other. Because EPCSs by definition bring the ER and PM into proximity with each other, we anticipate that any ER and any PM protein will co-localize to some extent. In this case, we included evidence that MSL10 and VAP27s can be at the same subcellular location only to support the split-ubiquitin and FRET-FLIM assays establishing that MSL10 and VAP27s interact. We added the subheading “A subpopulation of MSL10 co-localizes with a subpopulation of VAP27-1 and VAP27-3” to the results to better make this point.

5. While the localization of various markers is analysed in the msl10 mutant alleles, it appears that these are all static analyses, while EPCSs are not likely to be static. Did the authors address the dynamics of these markers in mutant backgrounds?

Based on this suggestion, we examined SYT1, SYT5, and SYT7 mobility in WT, *msl10-1* (loss-of-function) and *msl10-3G* (gain-of-function) backgrounds using FRAP. We did not find that *MSL10* had any obvious effect on SYT mobility (see Author response image 1). However, we note that there was a large degree of variability between plants of the same genotype. SYT1 mobility is reduced by salinity (Lee et al., 2019); perhaps it was also influenced by another environmental factor that we were not controlling for. The large degree of variation in our experiments below may have obscured a subtle effect of *MSL10* alleles on SYT mobility. As a result, we don’t feel these data to be a compelling addition to the manuscript and have not included them there.

**Author response image 1.** SYT1, SYT5, and SYT7 dynamics are not appreciably altered by MSL10. Fluorescence recovery after photobleaching (FRAP) curves for SYT1-GFP, SYT5-GFP, and SYT7-GFP in leaf epidermal cells of 4–5-week-old plants with the indicated *MSL10* genotypes. The number of individual plants examined is indicated above. The fluorescence intensity of the photobleached ROI was normalized to that of an unbleached, control ROI and by setting the initial fluorescence intensity to 100%. Error bars = SD.

6. While the data strongly suggest a role for MSL10 at EPCSs, this is not directly demonstrated, nor is the relevance of protein-protein interactions for MSL10 function shown. While this may be challenging, any analysis of the function of an MSL10 variant that does not localize to EPCSs or that can no longer interact with VAP27 or SYT1 would be very helpful in inferring such relevance.

We agree that this would be an excellent experiment. Unfortunately, such a variant does not exist, and all our efforts to create one have so far failed. For example, preliminary data suggest that the N-terminus of MSL10 is required for its interaction with VAP27 in the yeast two-hybrid (data not shown), but this domain is required for normal trafficking in plant cells, so we were unable to validate this in planta. We hope that the reviewers and editors will appreciate the leap forward in understanding MSL10 function provided by the current manuscript.

Reviewer #2 (Recommendations for the authors):

I very much appreciate the variety of approaches and methods and the description of rather interesting data.

Some comments for improvement of the manuscript:

1) The abstract could be more informative and detailed about the results presented in the work. Here I also would avoid "MSL10 can be found at EPSC" as I don't think this is really shown in the manuscript but rather indirect by interaction with VAP/SYT.

To avoid overstating our results, we have changed the text in the abstract to read “MSL10 associates with and functions with EPCS proteins.”

2) line 98: instead of WT (Col0) seedlings, should it be "microsomes"?

Yes, and it has been changed to “microsomes.”

3) In immunoprecipitation, a control using an unrelated/truncated plasma membrane protein and a GFP control would be advantageous over just a WT control. It would also be good to add the truncated list of 239 as a separate excel tab to source data 1.

We agree that including additional controls for co-IPs might have been useful, but we do not think the conclusions of this study would be considerably altered by including them. Perhaps different controls would have allowed us to exclude certain proteins from the 14 selected for further analysis if they, for example, interacted with GFP rather than MSL10. However, we were able to rule non-specific interactors out quite efficiently using yeast two-hybrid, and we did identify new and specific direct interactors of MSL10 using our approach.

We added the list of 239 selected proteins as a new tab in Figure 1- Source Data 1.

4) In the further analysis and comparison RTN3 and 6 are included as EPCS proteins for which I don't think there is any evidence. They might even obstruct the comparative approach here. Why are the VAPs not included in figure 1B?

We included RNTLB3/6 in our comparison based on their interaction with SYT1 and VAP27-1 in Kriechbaumer et al. (2015). Additionally, we consider plasmodesmata to be a specialized type of EPCS. However, to avoid over-interpretation we have changed the text to refer to RTNLB3/6 as “plasmodesmata-associated proteins” and “ER-shaping proteins found at plasmodesmata that interact with SYT1 and VAP27s”.

The VAP27s were not included in Figure 1B because a full proteome was not reported (Stefano et al., 2018). We have added a sentence in the figure legend to clarify this:

“The VAP27-1/3 interactome (Stefano et al., 2018) was not included here because only 8 selected interactors were reported.”.

5) The co-localisation of MLS10 and VAP27 (Figure 2D) should be analysed in cortical images.

Cortical images of tobacco epidermal cells have been added to show co-localization (Figure 3c).

6) In figure 3, the order of images does not correspond to the order of images called out in the text (g and d should be swopped). Adding text ("mapper" and "SYT") to B/C and E/F also would speed up the understanding of this figure to the reader. This is of course in the figure legend but this is quite extensive and it would be helpful in the figure as well.

Thank you for these suggestions. Images were rearranged in Figure 3 (which is now Figure 4) to follow the order they are mentioned in the main text. And labels for “UBQ:MAPPER-GFP,” “UBQ:VAP27-1-GFP”, and “UBQ:SYT1-GFP” were added to the figure for additional clarity.

7) A very brief summary of the data presented at the end of the results is always appreciated especially with an extensive dataset.

We have added a summary to the end of the Results section:

“In summary, in this study we identified three interactions between MSL10 and EPSCs: (1) a physical interaction between MSL10 and VAP27-1 and VAP27-3, (2) a functional interaction in which MSL10 promotes EPCS expansion, and (3) a genetic interaction in which mutations in SYT5 and SYT7 suppress MSL10’s signaling function.”
